# Effect of calcium on relieving berry cracking in grape (*Vitis vinifera* L.) ‘Xiangfei’

**DOI:** 10.7717/peerj.9896

**Published:** 2020-09-15

**Authors:** Jun Yu, Mingtao Zhu, Miao Bai, Yanshuai Xu, Shaogang Fan, Guoshun Yang

**Affiliations:** 1 Hunan Agricultural University, Changsha, China; 2 Hunan University of Humanities, Science and Technology, Loudi, China; 3 Engineering Research Center for Horticultural Crop Germplasm Creation and New Variety Breeding, Ministry of Education, Changsha, China

**Keywords:** *Vitis vinifera*, Calcium, Fruit cracking, Cell wall, RNA-Sequence

## Abstract

Fruit cracking is a physiological disorder in many plant species that leads to severe economic losses. The aim of this study was to investigate the effect of calcium on fruit cracking and explore the underlying mechanisms. We studied the effect of exogenous calcium on grape berry cracking, calcium absorbance and distribution, and cell wall metabolism in the cracking-susceptible cultivar ‘Xiangfei’. Calcium significantly reduced the frequency of fruit cracking, increased the break force of the berry skin, and stimulated storage of calcium. In addition, calcium increased the content of protopectin and inhibited the increase in content of water-soluble pectin, by regulating the transcription and activities of enzymes associated with cell wall metabolism. Taken together, the results indicated that dipping grape berries in calcium solution is effective in preventing fruit cracking by stimulating calcium uptake, inhibiting cell wall disassembly, and promoting cell wall strengthening.

## Introduction

Fruit cracking is a physiological disorder in which cracks appear in the fruit exocarp and mesocarp. This disorder affects the appearance of the fruit, increases water loss and susceptibility to pathogen infection, and decreases storage and shelf life, thus resulting in substantial commercial losses to growers (Ginzberg & Stern, 2016; Ramteke et al., 2017). Fruit cracking is influenced by external factors, including agronomic (irrigation, pruning, rootstock selection, and nutrition) and environmental factors (rainfall, temperature, and high humidity), and fruit characteristics, such as fruit shape, fruit size, sugar content, anatomy, and mechanical properties of the skin (Correia et al., 2018; Khadivi-Khub, 2014; Ramteke et al., 2017).

Cell wall disassembly, modification, and composition can affect the mechanical properties of the fruit pericarp and may be an important factor determining fruit susceptibility to cracking (Brüggenwirth & Knoche, 2017; Jiang et al., 2019; Yang et al., 2016). Cultivars that show a higher skin break force and elasticity are more resistant to fruit cracking (Brüggenwirth & Knoche, 2016; Huang et al., 2004; Yang et al., 2016). Cell wall metabolism is predominantly regulated by a suite of enzymes and proteins, including polygalacturonase (PG), expansin (EXP), pectinmethylesterase (PME), pectate lyase (PL), *β*-galactosidase (*β*-Gal), xyloglucan endotransglycosylase (XET), cellulose synthase (CES), and cellulase (Cx). Previous studies have reported that EXP, PG, *β*-Gal, and XET are closely associated with fruit cracking in tomato, litchi, apple, and sweet cherry (Balbontín et al., 2014; Kasai et al., 2008; Lu et al., 2006; Wang et al., 2006). In tomato fruit, the activities of PG, *β*-Gal, and Cx are distinctly higher in a cracking-susceptible genotype (Yang et al., 2016). At the gene level in tomato, suppression of a *β*-galactosidase gene (*TBG6*) increases the frequency of fruit cracking (Moctezuma, Smith & Gross, 2003) and suppression of *PG* expression results in decreased frequency of fruit cracking (Schuch et al., 1991). In sweet cherry and litchi, the expression level of EXP is significantly higher in cracking-resistant cultivars than in cracking-susceptible cultivars (Balbontín et al., 2014; Wang et al., 2006).

The fruit mineral nutrition status shows a strong relationship with fruit cracking. By analyzing the correlation between mineral element content and fruit cracking in 14 apricot cultivars, Nie et al. (2017) observed that calcium showed a strong negative correlation with fruit cracking (Nie et al., 2017). In litchi, the contents of calcium and boron in the pericarp were significantly higher in normal fruit than in cracked fruit (Li & Huang, 1996). The calcium content is significantly higher in a cracking-resistant cultivar and trees with a higher calcium content show a lower cracking rate compared with trees with a low calcium content (Huang et al., 1999; Li et al., 2001). In tomato, the calcium and boron contents are remarkably higher, whereas the potassium and magnesium contents are significantly lower in fruit of a cracking-resistant cultivar (Yang et al., 2016).

Calcium is an essential macroelement that performs a variety of important roles in plant physiology. Calcium is required for the structural integrity and stability of cell walls and membranes, and is involved in developmental and stress response signaling as a second messenger (Hashimoto & Kudla, 2011; Ranty et al., 2016; White & Broadley, 2003). In fruit trees, calcium is regarded as the most important nutrient determining fruit quality (Conway, Sams & Hickey, 2002). In grape, the berry calcium content does not increase after veraison owing to the nonfunctional xylem, low mobility of calcium in the phloem, and low transpiration rate of fruit (Knipfer et al., 2015; Rogiers et al., 2006; Rogiers et al., 2000). Therefore, to prevent localized calcium deficiency and promote fruit quality, it is necessary to supply calcium during the ripening stage. Preharvest applications of calcium-containing solutions decrease the incidence of fruit cracking in sweet cherry (Erogul, 2014; Michailidis et al., 2017), pomegranate (Davarpanah et al., 2018), and navel orange (Chen et al., 2014; Wen & Shi, 2012). However, the effect of calcium on grape berry cracking and its physiological and molecular mechanisms remain largely unexplored.

**Figure 1:**
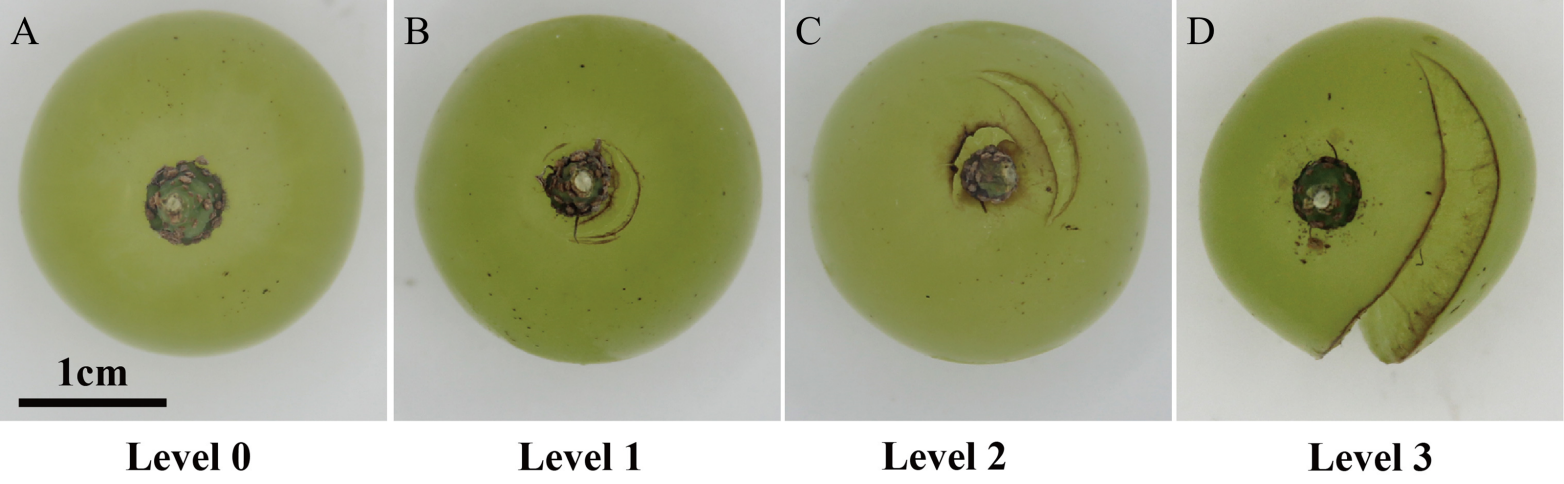
Fruit-cracking levels of grape berries based on severity. (A) Fruit-cracking level 0. (B) Fruit-cracking level 1. (C) Fruit-cracking level 2. (D) Fruit-crcacking level 3.

Table grape cultivars are grown in many countries worldwide and are popular with consumers owing to the color, aroma, taste, and nutritional content of the fruit (Ghan et al., 2017; Wang et al., 2017b). However, because of the high temperature and humidity in South China, the frequency of fruit cracking may be as much as 90% during the ripening stage in some grape cultivars, such as ‘Xiangfei’ (Fig. 1). Therefore, we studied the effect of exogenous calcium on grape berry cracking, skin mechanical properties, calcium absorbance and distribution, and cell wall metabolism. Based on the present results, potential mechanisms by which calcium reduces fruit cracking in grape are discussed.

## Material and Methods

### Plant material and calcium treatment

The experiment was conducted in 2017 and 2018 at the ‘Gan Shan’ experimental farm of Hunan Agricultural University, Changsha, Hunan province, China (latitude 28°11′N, longitude 113°4′E, elevation 43 m). Six-year-old plants of the table grape ‘Xiangfei’ grown under rain shelter were used. The plants were provided with drip irrigation, grown with inter-plant spacing of 2.8 m within rows and 1.8 m between rows, and trained to a “Y” form with each fruit cane bearing one cluster.

In 2017, to determine the optimal calcium concentration and application method for calcium treatment to inhibit fruit cracking, different calcium concentrations (0, 1.0, 3.0, 5.0, or 8.0 g L^−1^ CaCl_2_ ⋅ 2H_2_O containing 0.01% Tween 20 [v/v]) and application methods (foliar spray or fruit dipping) were applied in a preliminary trial. The percentage of cracked fruit in each cluster at the mature stage (73 days after full bloom [DAFB]) was recorded. Dipping fruit with 5 g L^−1^ solution at veraison was the most effective treatment to reduce the frequency of fruit cracking (Fig. S1). Therefore, this treatment was applied in a subsequent experiment performed in 2018.

In 2018, grape clusters were dipped in 5 g L^−1^ CaCl_2_ ⋅ 2H_2_O (purity: 98%) solution containing 0.01% Tween 20 (v/v) at veraison (8 June, 40 DAFB). The control clusters were dipped in deionized water containing 0.01% Tween 20 (v/v). All clusters were immersed for 2 min and thereafter allowed to dry on the vine. Both treatments were conducted on a sunny morning from 06:00 to 07:00 (temperature range: 26–30 °C). The treatment and control were performed with three biological replications (three vines per replication). Identical irrigation and fertilization protocols were applied to both treatments. Samples of the fruit skin were collected at 0 weeks (40 DAFB), 1 week (47 DAFB), 2 weeks (54 DAFB), and 3 weeks (61 DAFB) after treatment for histological observation, and determination of calcium content, cell wall polysaccharide components, and cell wall-associated enzyme activities. For each replication, five clusters were mixed, the fruit were peeled immediately, snap frozen, ground to powder, and stored at −80 °C in an ultra-low temperature refrigerator for further analysis.

### Quality analysis

At the commercial harvest stage (75 DAFB), 30 berries (10 berries per replication) from different positions throughout the clusters were randomly collected for measurement of fruit quality traits, namely vertical fruit diameter, transverse fruit diameter, total soluble solids (TSS), titratable acidity, and water content. The vertical diameter and transverse diameter were measured using an electronic caliper. Titratable acidity was expressed as grams tartaric acid per liter and was quantified by titrating 10 mL of juice with 0.1 N NaOH to pH 8.1 (Ortiz, Graell & Lara, 2011; Wang et al., 2017a). Total soluble solids content was measured with a digital refractometer at room temperature (Xu et al., 2016). To determine the water content, the whole berry was oven-dried to constant weight at 60 °C (Yang et al., 2016). The percentage water content of the berry was calculated using the formula (fresh weight–dry weight)/fresh weight ×100.

### Measurement of environmental variables

Environmental variables were recorded at hourly intervals using an Em50 series data logger (Decagon Devices, Inc., Pullman, WA, USA). The VP-4 sensor was used to measure air temperature and relative humidity. The EC-5 sensor was used to measure soil moisture content.

### Evaluation of fruit cracking

Fruit cracking incidence (%) was recorded for each cluster and was calculated as the number of cracked berries/total berries in each cluster ×100. For each replication, 10 clusters were randomly selected from three vines and visually examined for cracks on the fruit surface. The mean of the three replications represented the percentage of cracked fruit.

Cracks in each fruit were evaluated on the basis of a severity index using a scale of 0 to 3, where 0 = no cracks, 1 = cracks less than one cm in length, 2 = cracks 1–2 cm long, and 3 = cracks more than two cm in length (Fig. 1). The number of cracked berries of each severity (N0 to N3) and the total berry number in a cluster (Nt) were recorded (Cline et al., 1995; Lichter et al., 2002).

The cumulative severity index was calculated using the following formula:

\begin{eqnarray*}\text{Cumulative severity index}= \frac{ \left( \mathrm{N}0\ast 0+\mathrm{N}1\ast 1+\mathrm{N}2\ast 2+\mathrm{N}3\ast 3 \right) \ast 100}{\mathrm{Nt \ast }3} \end{eqnarray*}

### Measurement of mineral element contents in the skin

The fruit used for mineral analysis were washed twice with 1% (w/v) citric acid and then rinsed with deionized water three times. The skin samples were oven-dried at 105 °C and ashed in a muffle furnace at 550 °C. A sample (0.1 g) of ash was digested with 10 mL HNO_3_ using a microwave digestion system (MARS, CEM Corporation, Matthews, NC, USA). The contents of the mineral elements calcium, potassium, magnesium, and boron were determined using an inductively coupled plasma–optical emission spectrometer (Optima 8300, Perkin Elmer, Shelton, CT, USA) (Lu et al., 2013).

### Break force of the berry skin

**Figure 2:**
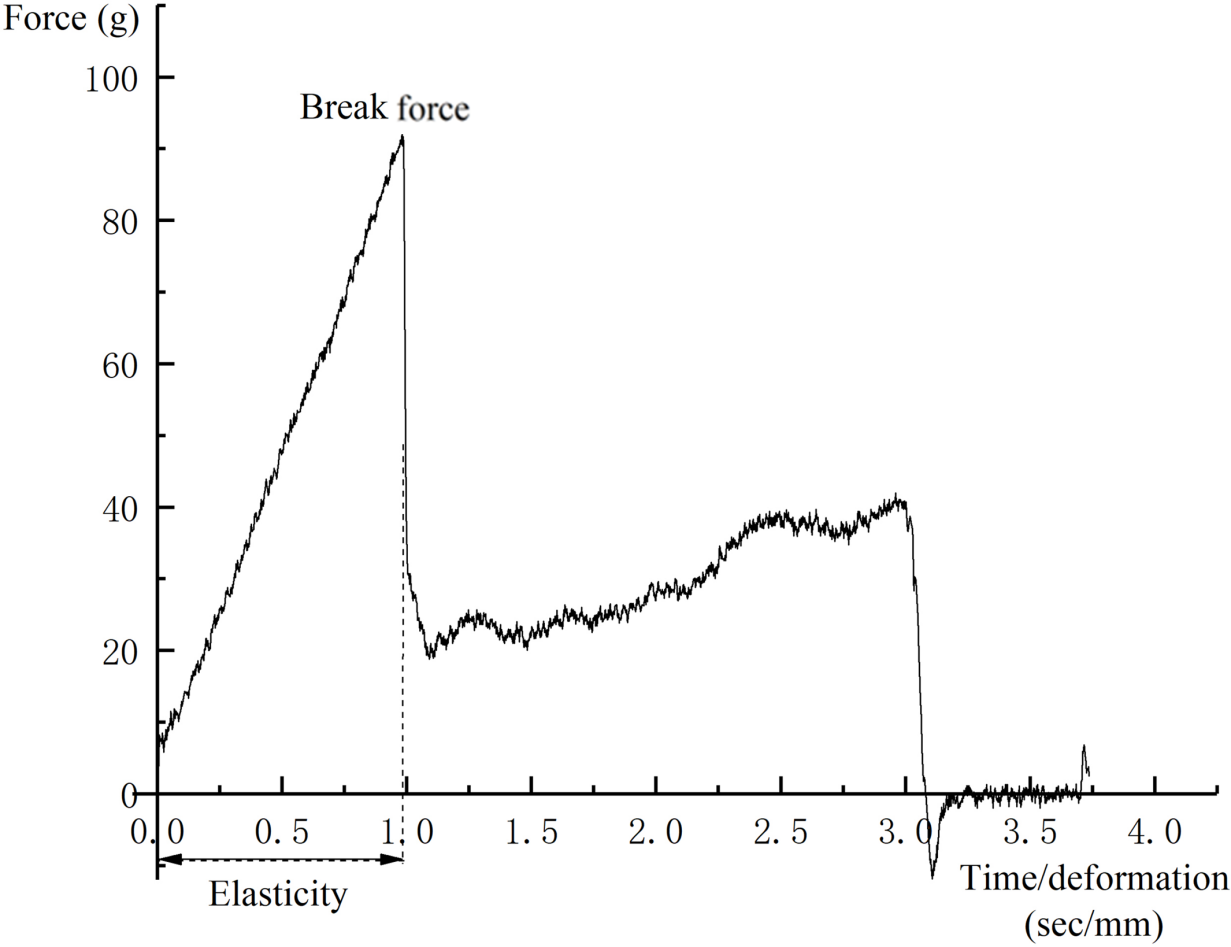
A typical force-deformation curve of puncture test.

Three grape berries were selected from each replication and the break force (g) was measured using a TA.XTplus Texture Analyzer (Stable Micro Systems, Surrey, UK). A puncture test was conducted by compressing the fruit at the equator with a P/2N needle to a depth of three mm at the rate of one mm s^−1^ (Letaief et al., 2008; Zsófi et al., 2014). A typical force–deformation curve is presented in Fig. 2. The maximum force required to puncture the skin was considered to be the skin break force.

### Cytochemical observation of Ca^2+^ localization

The pericarp tissue in the equator was excised with a razor blade and immediately fixed with 3% glutaraldehyde in 0.1 M potassium phosphate buffer (pH 7.2) containing 2% potassium antimonate at 4 °C for 24 h. After rinsing three times, the sample was post-fixed, dehydrated, and embedded in epoxy resin (Tonetto de Freitas et al., 2011). Thin sections (70 nm thick) were cut using an ultramicrotome (EM UC6, Leica, Mannheim, Germany) and stained (Dong et al., 2015). To confirm whether the precipitate was Ca^2+^-pyroantimonate, a control section was immersed in 100 mM ethylene glycol tetraacetic acid (EGTA; pH 7.9) and rinsed with distilled water (Tonetto de Freitas et al., 2011). The sections were observed using a transmission electron microscope (H-7500, Hitachi, Tokyo, Japan) at an acceleration voltage of 80 kV. The area of five cells was measured using ImageTool 2.0 software. The number of calcium grains was counted in the same five cells and the number of calcium grains per 1 µm^2^ was calculated.

### Microscopic observation of the fruit surface

Berry pericarp samples (3 mm ×3 mm) at the equator were excised to observe histological changes in the berry surface. The samples were fixed and dehydrated in accordance with the method of Inchang et al. (2007). The samples were desiccated in a critical point dryer (HCP-2, Hitachi, Japan), sputter-coated with platinum with an auto fine coater (JFC-1600, JEOL, Tokyo, Japan), and viewed with a scanning electron microscope (JSM-6380LV, JEOL, Japan).

### Energy dispersive X-ray mapping of calcium

The fruit were washed twice with 1% (w/v) citric acid and then rinsed with deionized water three times. Using a previously described method (Fernando et al., 2008), cross-sectional skin samples were excised from a berry, immediately frozen in liquid nitrogen, and freeze-dried for 48 h at 0.3 mba and −45 °C (Alpha 1-4/LSC Plus, Martin Christ, Osterode am Harz, Germany). After platinum coating, the samples were observed with a scanning electron microscope (JSM-6701F, JEOL, Japan) and analyzed for calcium distribution using an energy dispersive spectrometer (JSM-6701F, JEOL, Japan).

### Analysis of cell wall components

The cell wall materials were isolated from the berry skin. A sample (200 mg) of the isolated cell wall materials was fractionated into water-soluble pectins (WSP), CDTA-soluble pectins (CSP, ionically bound pectic polysaccharides), sodium carbonate-soluble pectins (SSP, covalently-bound pectic polysaccharides), hemicellulose, and cellulose Deng, Wu & Li (2005). Uronic acid contents of the pectin fractions were measured by the meta-hydroxydiphenyl method using galacturonic acid as the standard (Blumenkrantz & Asboe-Hansen, 1973). The cellulose and hemicellulose fractions were quantified by the anthrone method using glucose as the standard (Vicente et al., 2005).

### Cell wall-modifying enzyme activity assays

A crude enzyme extract was prepared in accordance with the method described by Deng, Wu & Li (2005). The activity of PME was determined by titrating five mL of 1% (w/v) citrus pectin solution, which was adjusted to pH 7.5 with 0.1 M NaOH. After addition of 0.5 mL crude enzyme extract, the reaction mixture was stirred continuously at 35 °C for 30 min, and the mixture pH was maintained at 7.5 with 0.01 M NaOH. The volume of 0.01 M NaOH consumed was recorded. One unit (U) of activity was expressed as 1 µmol NaOH consumed per gram fresh weight (FW) per minute (Wegrzyn & MacRae, 1992).

The activity of PG was measured as described by Balic et al. (2014) with minor modifications. The reaction mixture of one mL sodium acetate buffer (50 mM, pH 5.5), 0.5 mL polygalacturonic acid (1%, w/v), and 0.5 mL crude enzyme extract was incubated at 37 °C for 1 h. To measure the amount of reduced sugars, 1.5 mL 3,5-dinitrosalicylic acid (DNS) was added. After boiling for 5 min and then cooling, the absorbance was measured at 540 nm. Glucose was used as the standard. One unit (U) of activity was defined as 1 µmol reducing sugar released per gram FW per minute.

Activity of *β*-Gal was assayed as described by Wei et al. (2010). First, 0.5 mL of 3 mM *p*-nitrophenyl-*β*-D-galactopyranoside and 0.5 mL sodium acetate buffer (0.1 M, pH 5.2) were pre-incubated at 40 °C for 10 min, followed by addition of 0.5 ml crude enzyme extract. After incubation at 37 °C for 30 min, the reaction was terminated by addition of 2.0 mL of 0.5 M sodium carbonate. The released *p*-nitrophenyl was quantified against a standard curve for *p-* nitrophenol measured at 400 nm. One unit (U) of *β*-Gal was defined as the liberation of 1 nmol *p*-nitrophenol per gram FW per minute.

Cellulase activity was measured using the DNS method with carboxymethylcellulose (CMC) as the substrate (Deng, Wu & Li, 2005). A mixture of 1.5 mL CMC (1% [w/v] in 50 mM sodium citrate buffer, pH 5.0) and 0.5 mL crude enzyme extract was incubated at 37 °C for 1 h, then 1.5 mL DNS was added to the mixture. After heating at 100 °C for 5 min, the amount of reducing sugar was determined spectrophotometrically at 540 nm with glucose used as the standard. One unit (U) of activity was expressed as 1 µmol reducing sugar per gram FW per minute. All assays were conducted with boiled crude enzyme extract as the control.

### RNA sequencing analysis

Berry skin samples collected at 1 week (CK1 and Ca1), 2 weeks (CK2 and Ca2), and 3 weeks (CK3 and Ca3) after calcium treatment were used for RNA sequencing (RNA-Seq). Berry skin samples from the three replications were combined prior to RNA extraction. Total RNA was extracted from frozen samples with TRIzol^®^ Reagent (Invitrogen, Carlsbad, CA, USA). Six RNA-Seq libraries were constructed using the TruSeq™ RNA Sample Preparation Kit (Illumina, San Diego, CA, USA) in accordance with the manufacturer’s protocol. The libraries were sequenced using an Illumina HiSeq X Ten system by the Shanghai Majorbio Bio-pharm Biotechnology Co. (Shanghai, China) to obtain 150 bp paired-end reads. The reads were mapped to the 12X.2 version of the *Vitis vinifera* ‘PN40024’ reference genome using TopHat software (Trapnell, Pachter & Salzberg, 2009). All RNA-Seq raw data files were uploaded to the NCBI SRA database (accession numbers SRR11531447, SRR11531448, SRR11531449, SRR11531450, SRR11531451, and SRR11531452). The expression level of each transcript was determined by calculating fragments per kilobases per million reads (FPKM) with RSEM software (Li & Dewey, 2011). A value FPKM >5 was used as the stringent cutoff to identify expressed genes (Dong et al., 2013). The genes that showed significant differential expression between the calcium treatment and control were determined by comparing the raw counts of each transcript using DESeq2 software (Varet et al., 2016). Genes with *p-* adjust <0.05 and —log_2_ fold change— ≥ 1 were defined as significantly differentially expressed genes (DEGs). The genes that showed a significant differential expression value for at least one time point were selected for further analysis.

## Results

### Effect of calcium on berry development

**Figure 3:**
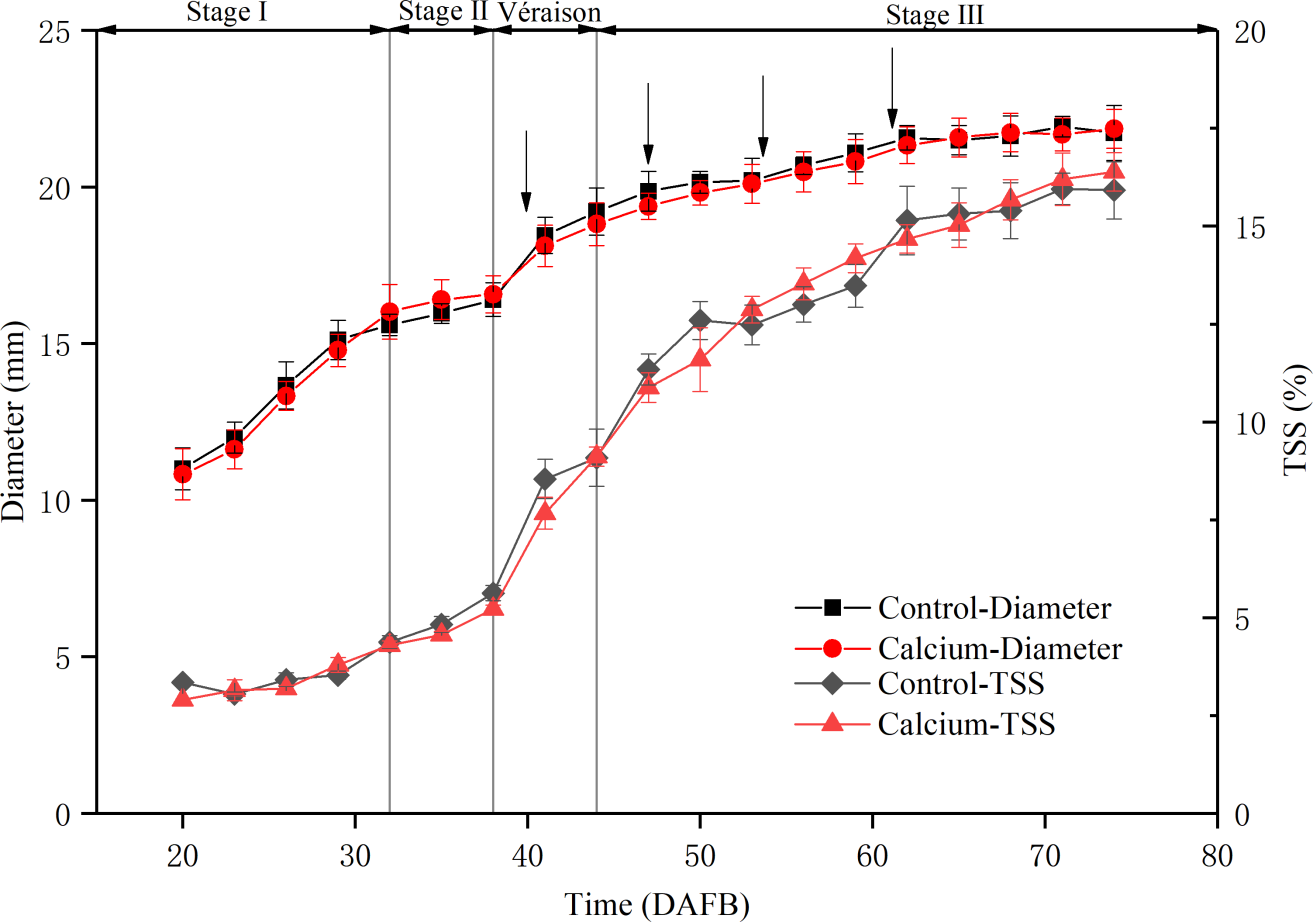
Fruit diameter and total soluble solids content during development of grape ‘Xiangfei’ berries in calcium treatment and control. Black arrows indicate the sampling dates. Error bar stands for standard deviation (SD).

To assess the physiological stage of the grape berry, the fruit diameter and total soluble solids content were measured during development (Fig. 3). The berry diameter displayed a typical double sigmoid curve. The first rapid growth stage (stage I) was initiated from fruit set to about 32 DAFB. Stage II comprised a lag phase, from 32 to 38 DAFB, when the berry diameter changed little and sugars started to accumulate. Veraison, from 38 to about 44 DAFB, marked initiation of the second rapid growth stage, texture softening, and rapid sugar accumulation. During the middle and late stage III, the total soluble solids content increased sharply until 75 DAFB. During the entire developmental period, no significant difference was observed in fruit diameter and total soluble solids content between the calcium treatment and control, which indicated that calcium treatment did not delay fruit ripening.

Berry quality traits were measured at the commercial harvest stage (75 DAFB). Preharvest calcium treatment did not significantly affect fruit size, soluble sugars content, titratable acidity, and water content of the berries at harvest (Table 1), which indicated that calcium treatment did not influence the fruit quality.

**Table 1:** Fruit qualities of grape berry under calcium treatment.

	**Calcium**	**Control**
Longitudinal diameter (mm)	23.29 ± 1.10a	22.15 ± 0.63a
Transverse diameter (mm)	21.85 ± 0.71a	21.72 ± 0.59a
Total soluble solid (%)	16.38 ± 0.11a	15.91 ± 0.29a
Titratable acidity (%)	0.29 ± 0.02a	0.36 ± 0.04a
Water content (%)	83.16 ± 0.59a	82.27 ± 0.26a

**Notes.**

The same letter represents no significant difference between calcium and control according to Duncan’s multiple range test at *P* < 0.05.

### Effect of calcium on fruit cracking

**Figure 4:**
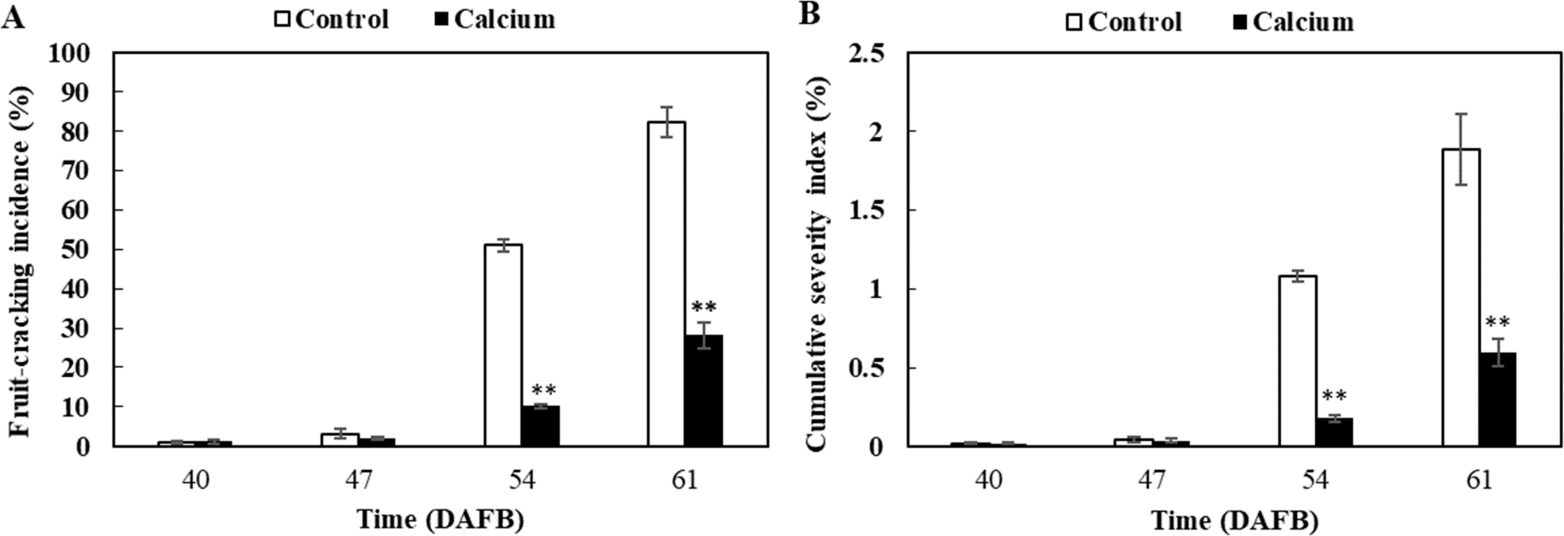
Fruit-cracking incidence (A) and cumulative severity index (B) of grape berry treated with calcium. Error bar stands for standard deviation (SD). Asterisks “*” and “**” indicate significant difference between calcium and control at *P* < 0.05 and *P* < 0.01 level, respectively.

No significant difference in fruit-cracking incidence and severity index was observed between the calcium treatment and control at 47 DAFB (Fig. 4). However, fruit-cracking incidence and severity index increased sharply at 54 DAFB and 61 DAFB in the calcium treatment and the control, which may be attributed to the sudden changes in soil moisture content, air temperature, and relative humidity resulting from heavy rainfall during the second week after calcium treatment (Fig. S2). In addition, the fruit-cracking incidence in the calcium treatment was significantly lower than that of the control (40.88% and 54.15% lower at 54 and 61 DAFB, respectively) (Fig. 4A). Similar to fruit-cracking incidence, at 54 and 61 DAFB, the cumulative severity index in the calcium treatment was significantly lower (about 0.9% and 1.3% lower at 54 and 61 DAFB, respectively) compared with that of the control (Fig. 4B).

### Microscopic observation of berry surface

**Figure 5:**
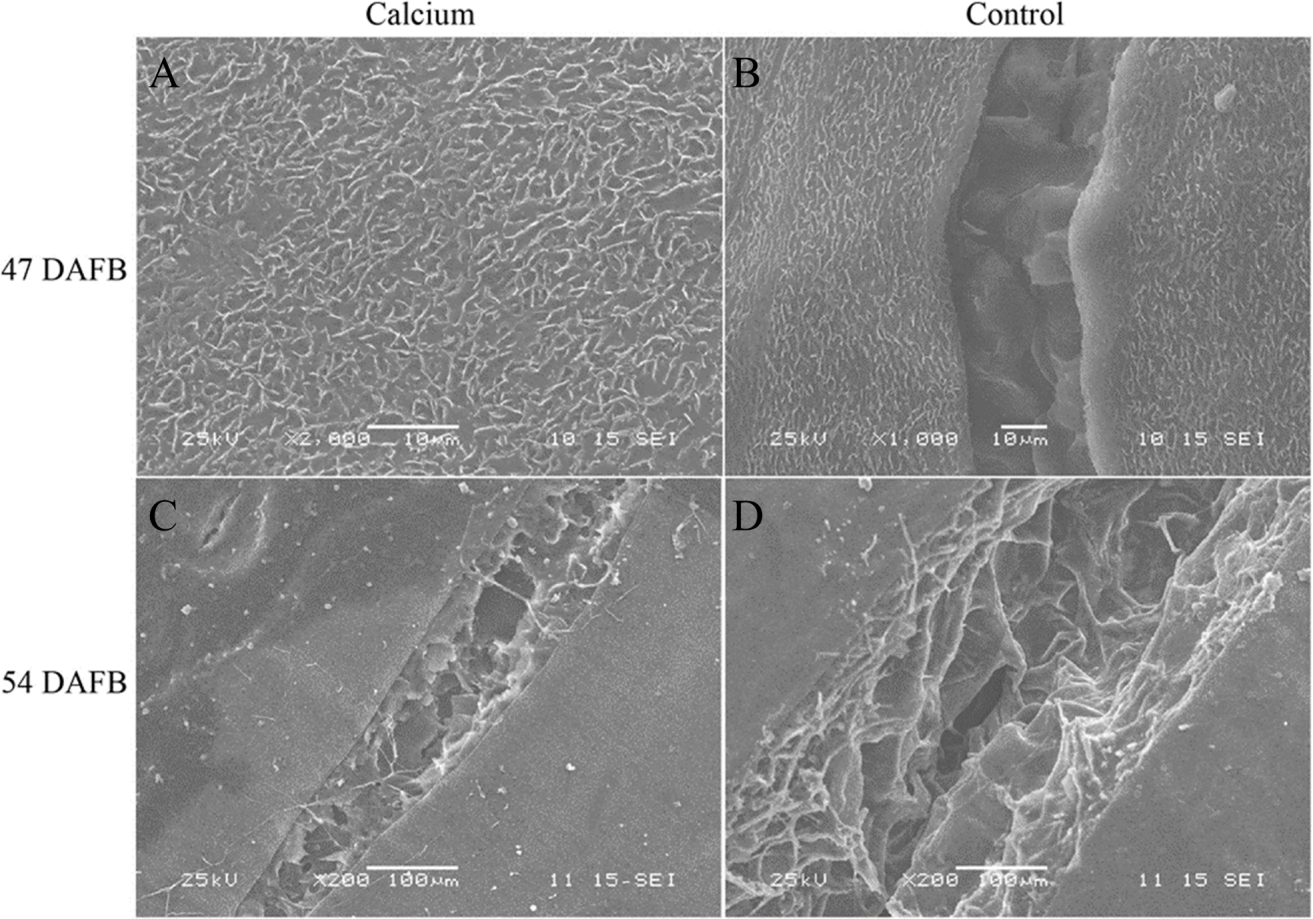
Micrographs of fruit surface observed by scanning electron microscopy. (A) Fruit surface of calcium treament at 47 DAFB. (B) Fruit surface in control at 47 DAFB. (C) Fruit surface of calcium treament at 54 DAFB. (D) Fruit surface in control at 54 DAFB.

At 47 DAFB, little macrocracks were observed on the fruit surface in the calcium treatment and the control. Concentric fine cracks, which were limited to the cuticle and epidermal cells, had developed on the base of pedicel on berry skin in the control, whereas the majority of berries in the calcium treatment were free of concentric fine cracks. At 54 DAFB, large and macroscopically visible cracks were developed in fruit of the calcium treatment and control. The cracks in control fruit were wider and deeper compared with those of the calcium treatment. The cracks in control fruit extended deeply almost into the mesocarp, whereas in the calcium treatment cracks were confined to the outermost cell layers (Fig. 5).

### Effect of calcium on mechanical properties of the berry skin

**Figure 6:**
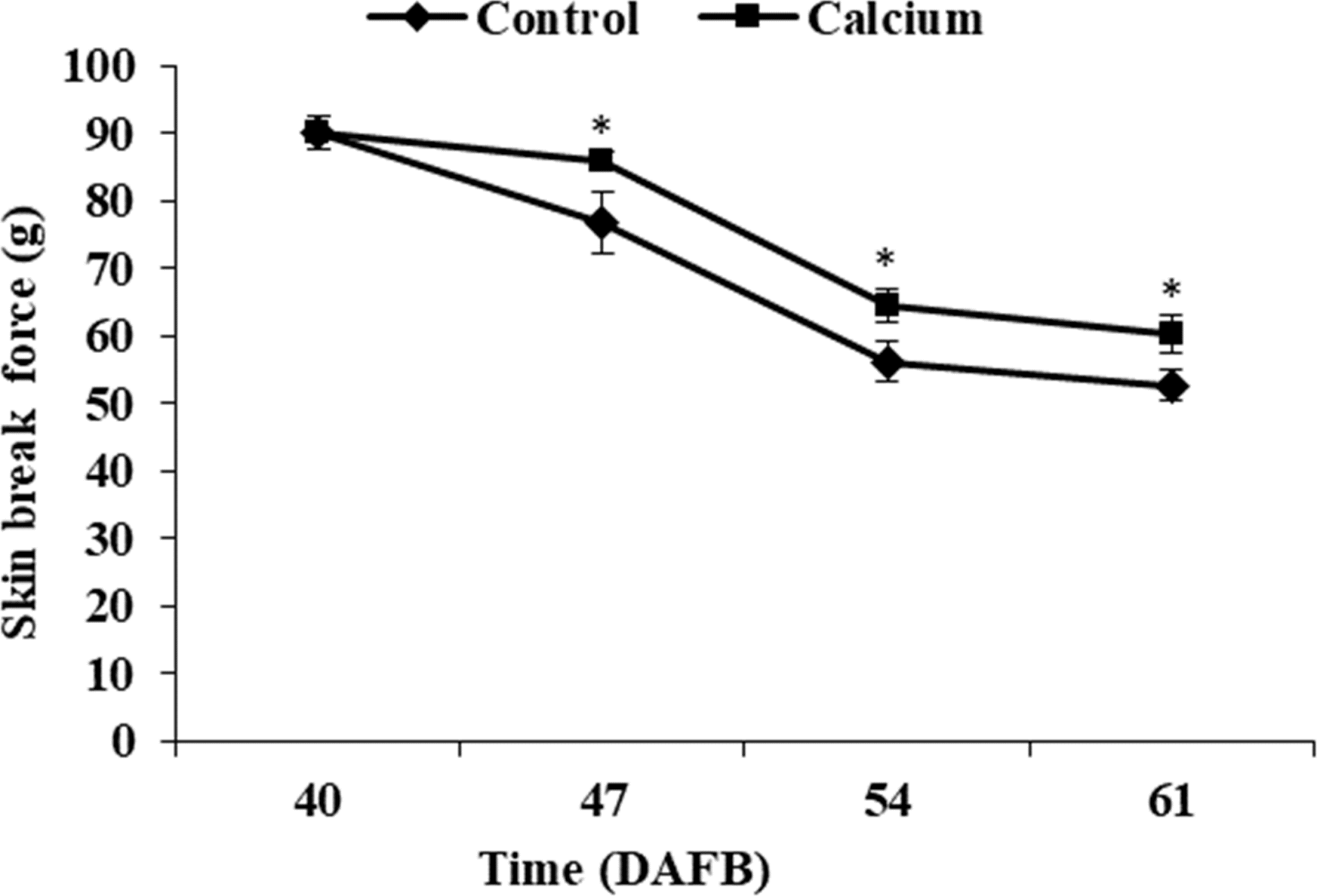
Break force of grape skin under calcium treatment. Error bar stands for standard deviation (SD). Asterisks “*” and “**” indicate significant difference between calcium and control at *P* < 0.05 and *P* < 0.01 level, respectively.

The berry skin break force in the calcium treatment and control decreased progressively as the fruit matured. The break force of the calcium treatment was significantly higher than that of the control at the same time point from 47 to 61 DAFB (Fig. 6).

### Effect of calcium on mineral nutrient contents of the berry skin

**Figure 7:**
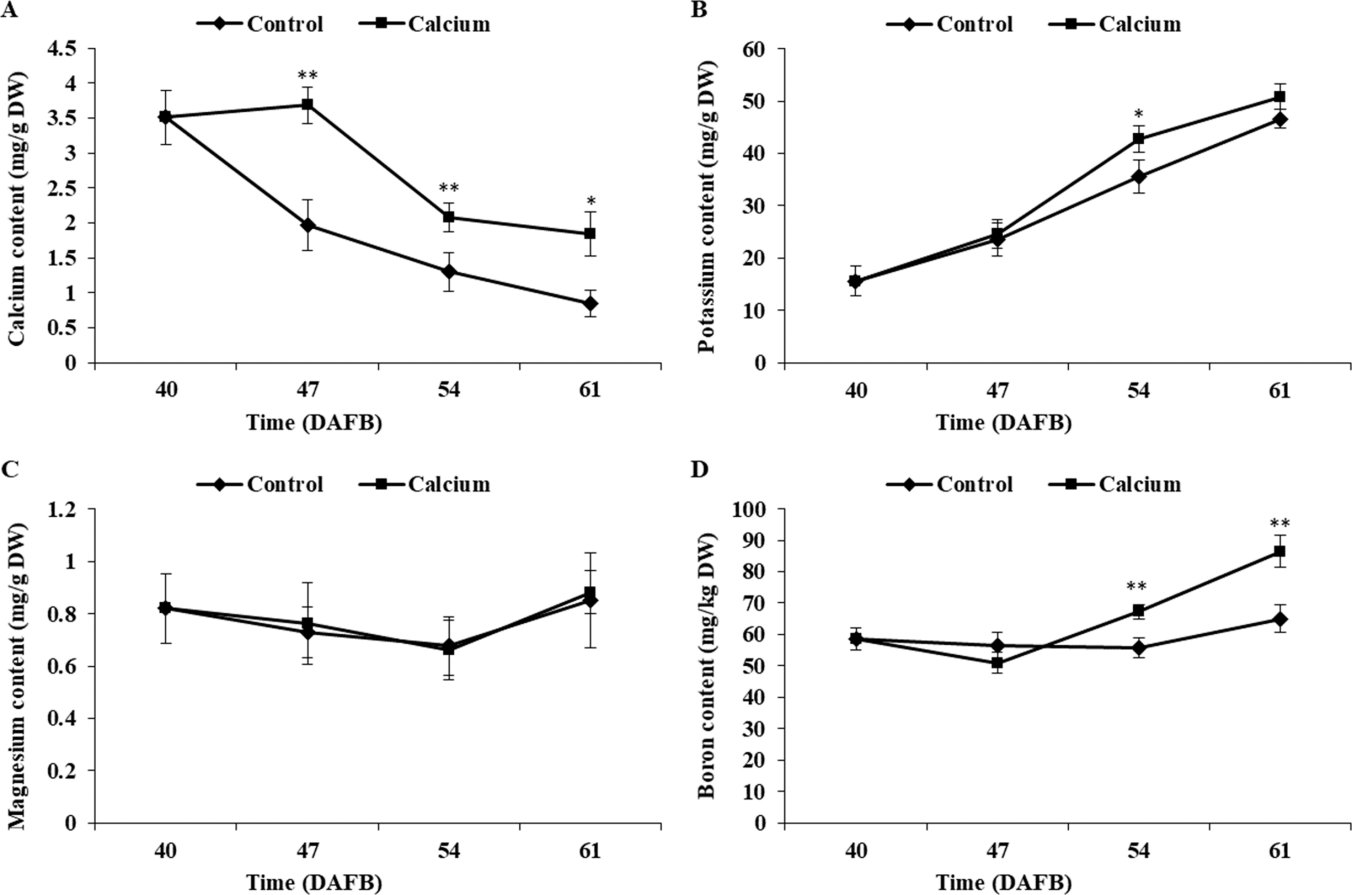
The Ca, K, Mg, B content of grape skin under calcium treatment. Error bar stands for standard deviation (SD). Asterisks “*” and “**” indicate significant difference between calcium and control at *P* < 0.05 and *P* < 0.01 level, respectively.

**Figure 8:**
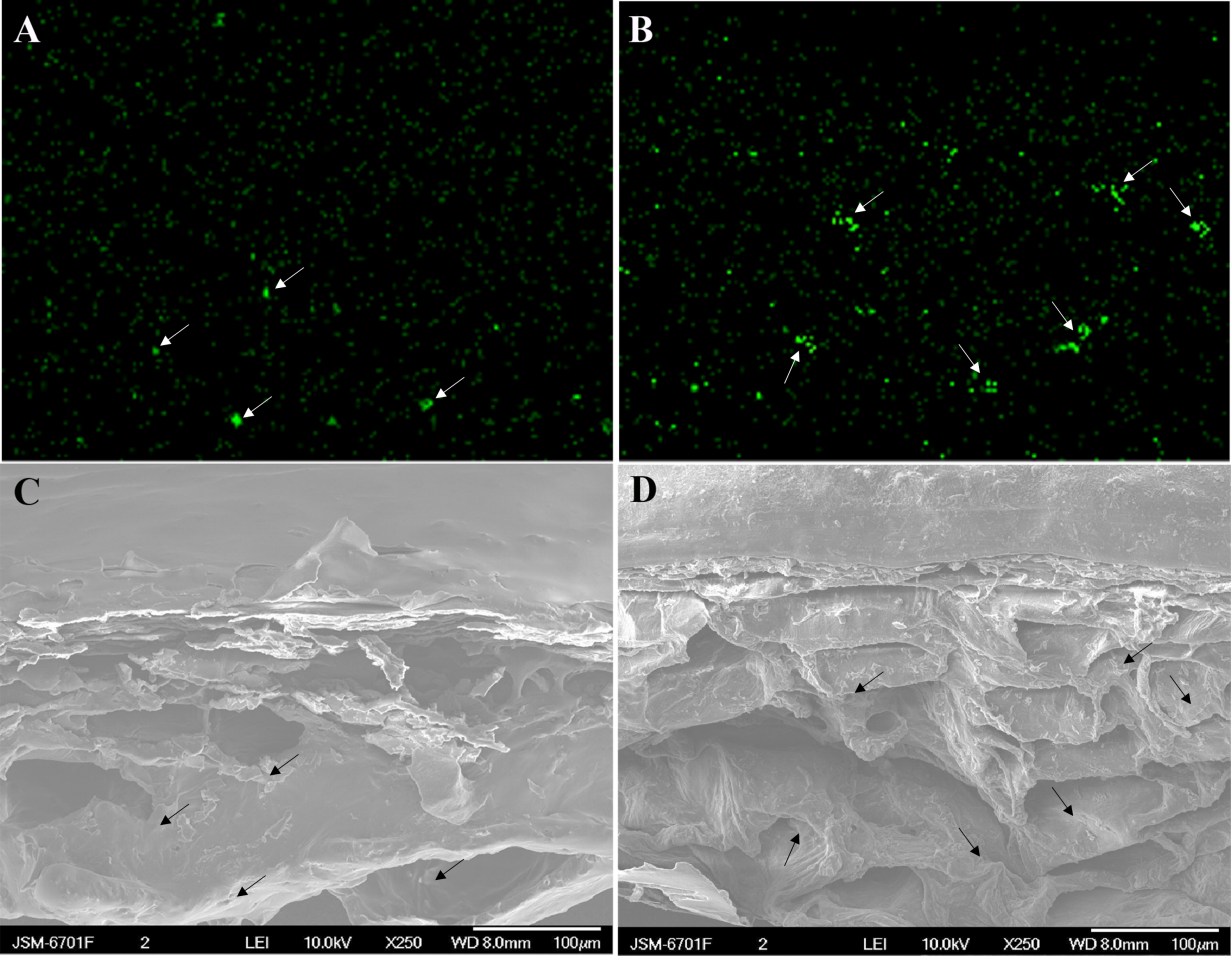
Cross-sectional element mapping and corresponding scanning electron micrograph of the skin of grape ‘Xiangfei’ berries at 47 days after full bloom. Green fluorescence indicates calcium-rich zones. The corresponding calcium-rich zone in the cellular image is indicated by arrows. Control: (A) and (C); Calcium: (B) and (D).

Atomic absorption spectrometry revealed that the total calcium content of the berry skin in the calcium treatment showed a slight rise followed by a decline during fruit development, whereas the calcium content in the control decreased sharply during the ripening stage. The total calcium content of the berry skin in the calcium treatment was significantly higher compared with that of the control from 47 to 61 DAFB (Fig. 7). Mapping of calcium distribution detected more calcium-rich zones in the berry skin in the calcium treatment than in the control at 47 DAFB (Fig. 8), which was consistent with the total calcium contents.

The potassium content increased remarkably during ripening in both treatments. The potassium content in the calcium treatment was generally higher than that of the control with a significant difference observed at 54 DAFB (Fig. 7).

The boron content in the control showed little change during ripening, whereas in the calcium treatment the boron content increased sharply after a slight decrease at 47 DAFB. The boron content in the calcium treatment was significantly higher than that of the control at 54 and 61 DAFB (Fig. 7).

The magnesium content changed little during ripening in both treatments. No significant difference between the calcium treatment and control was observed for all time points (Fig. 7).

### Effect of calcium on cellular calcium distribution in the berry skin

**Figure 9:**
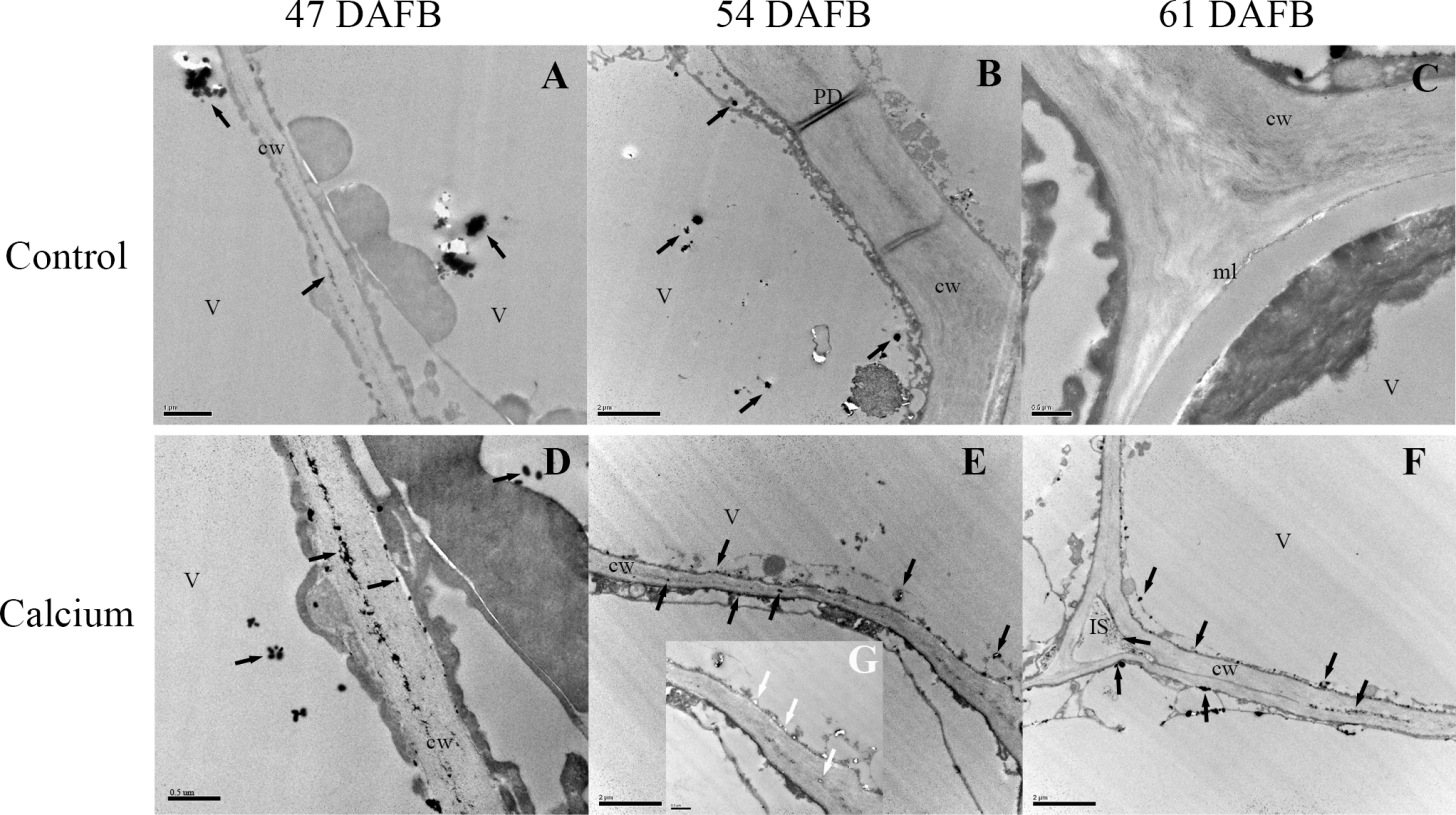
Cellular distribution of calcium in cell wall of epidermal and hypodermal cells. Control at 47 DAFB (A), 54 DAFB (B)and 61 DAFB (C); Calcium treatment at 47 DAFB (D), 54 DAFB (E)and 61 DAFB (F). Black arrows indicate Ca grains. White arrows indicate the Ca grains washed by EGTA. cw, cell wall; V, vacuole; IS, intercellular space; PD, plasmodesmata; ml, middle lamella.

**Figure 10:**
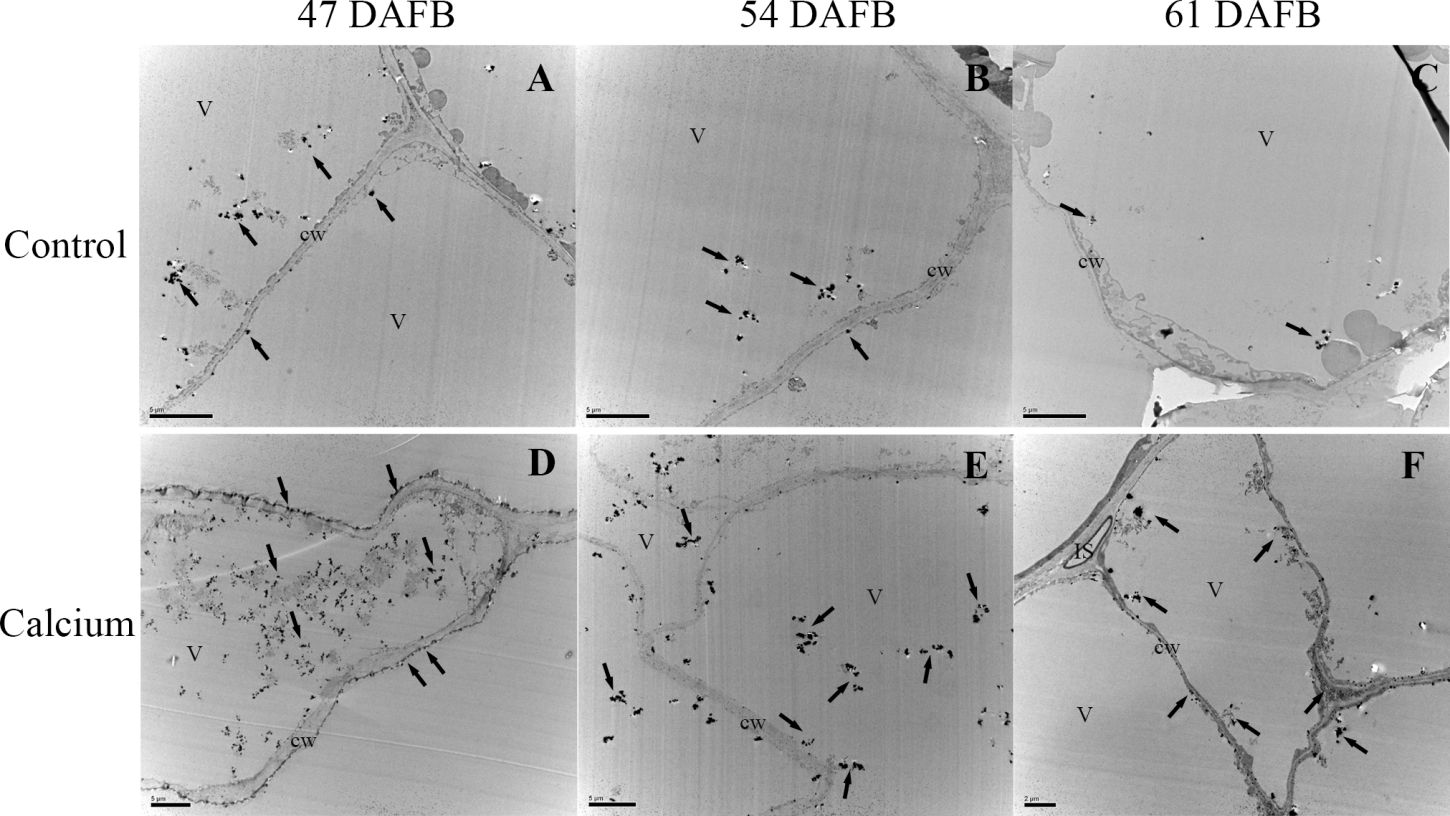
Cellular distribution of calcium in vacuole of epidermal and hypodermal cells. Control at 47 DAFB (A), 54 DAFB (B)and 61 DAFB (C); Calcium treatment at 47 DAFB (D), 54 DAFB (E) and 61 DAFB (F). Black arrows indicate Ca grains. cw, cell wall; V, vacuole; IS, intercellular space.

**Figure 11:**
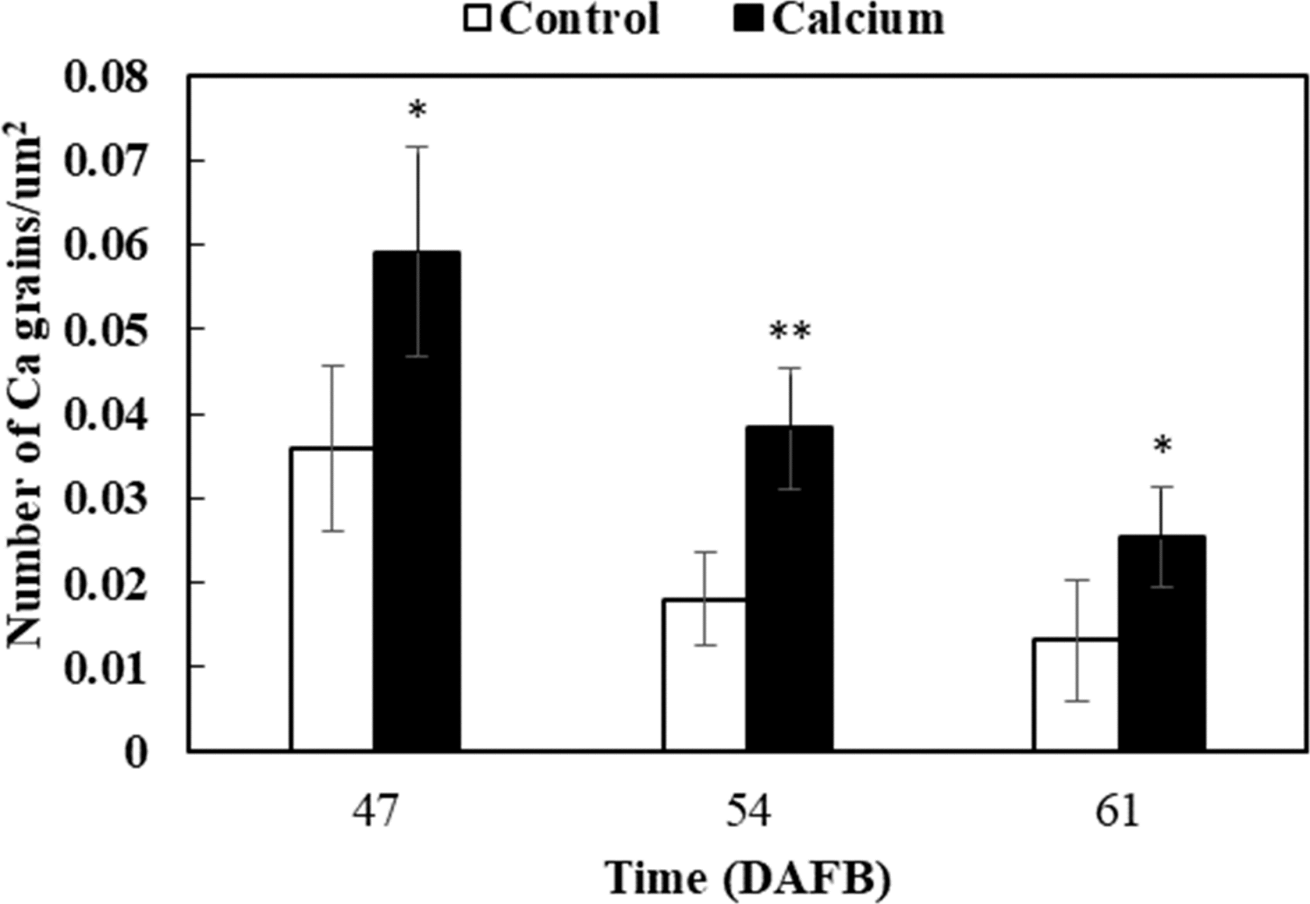
Accumulation of calcium grains/um^2^ in the skin of grape ‘Xiangfei’ berries after calcium treatment. Error bar stands for Standard deviation (SD). Asterisks “*” and “**” indicate significant difference between calcium and control at *P* < 0.05 and *P* < 0.01 level, respectively.

After dipping fruit in calcium solution, the calcium grains mainly accumulated in the cell wall and vacuole of epidermal and hypodermal cells (Figs. 9 and 10). Although the amount of calcium grains decreased distinctly during ripening, more calcium grains accumulated in the calcium treatment compared with that of the control (Fig. 11). To prove the presence of calcium grains, the black grains were removed by chelation with EGTA solution, labelled with white arrows (Fig. 9G).

### Effect of calcium treatment on cell wall polysaccharide components in the berry skin

In control fruit, WSP contents increased 1.93-fold from 40 to 64 DAFB, whereas CSP and SSP contents decreased to 54% and 41% of the initial values. The calcium-treated fruit showed a distinctly delayed increase in WSP content and decreases in CSP and SSP contents. The WSP content was higher, and the CSP and SSP contents were lower, in the calcium treatment compared with those of the control. The total pectin content decreased continuously from 47 DAFB. At all three time points, the total pectin content in the calcium treatment was higher than that of the control.

**Figure 12:**
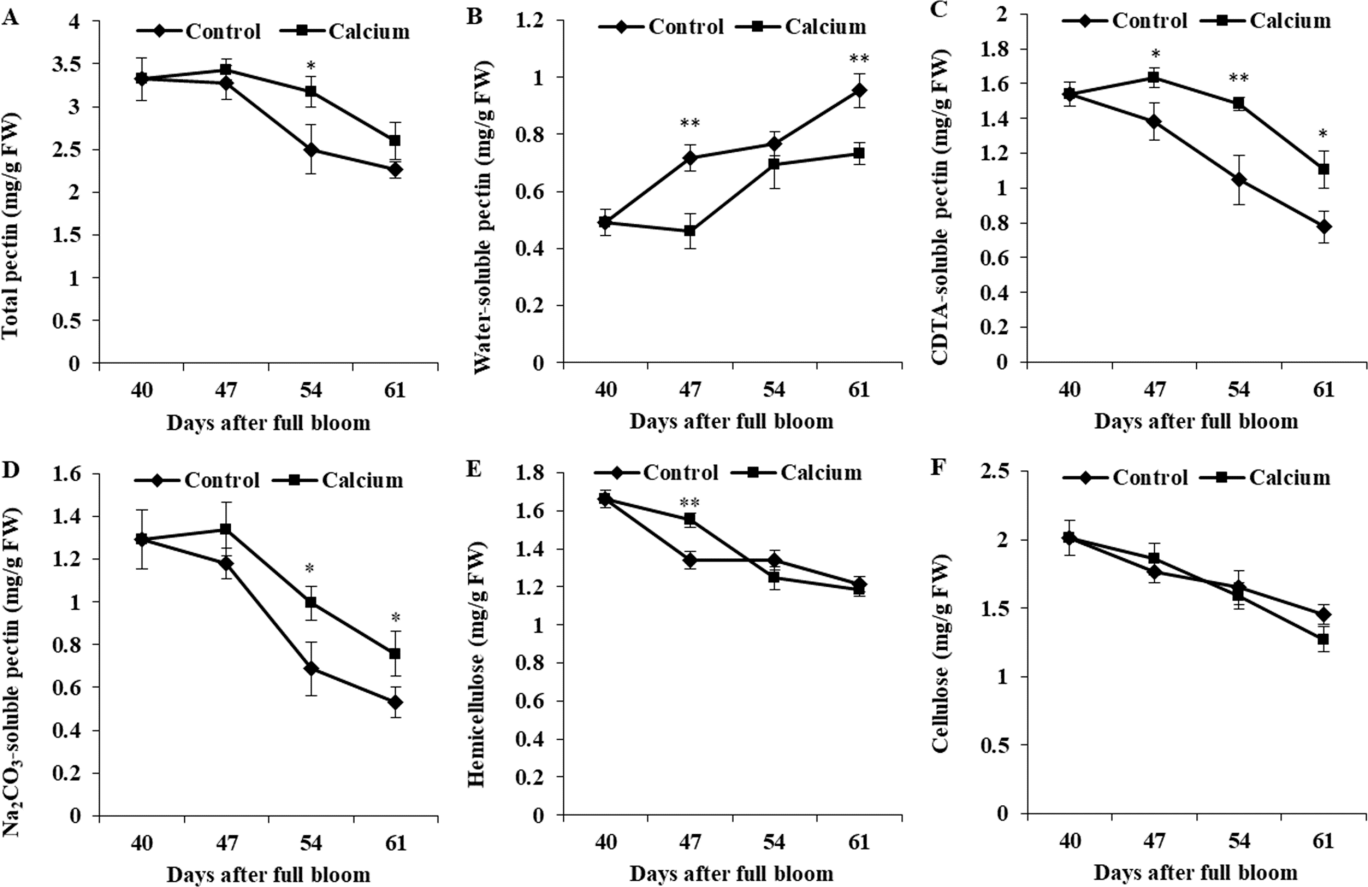
Cell wall polysaccharides component of treated and untreated grape skin. (A) Total pectin. (B) Water-soluble pectin. (C) CDTA-soluble pectin. (D) Na_2_CO_3_-soluble pectin. (E) Hemicellulose. (F) Cellulose. Error bar stands for standard deviation (SD). Asterisks “*” and “**” indicate significant difference between calcium and control at * P* < 0.05 and *P* < 0.01 level, respectively.

The hemicellulose and cellulose contents of both treatments were reduced during ripening. For hemicellulosic polysaccharides, calcium only significantly relieved the degradation at 47 DAFB. No significant difference in cellulose content between the calcium treatment and control was observed at the same time point (Fig. 12).

### Effect of calcium on cell wall-associated enzyme activities in the berry skin

**Figure 13:**
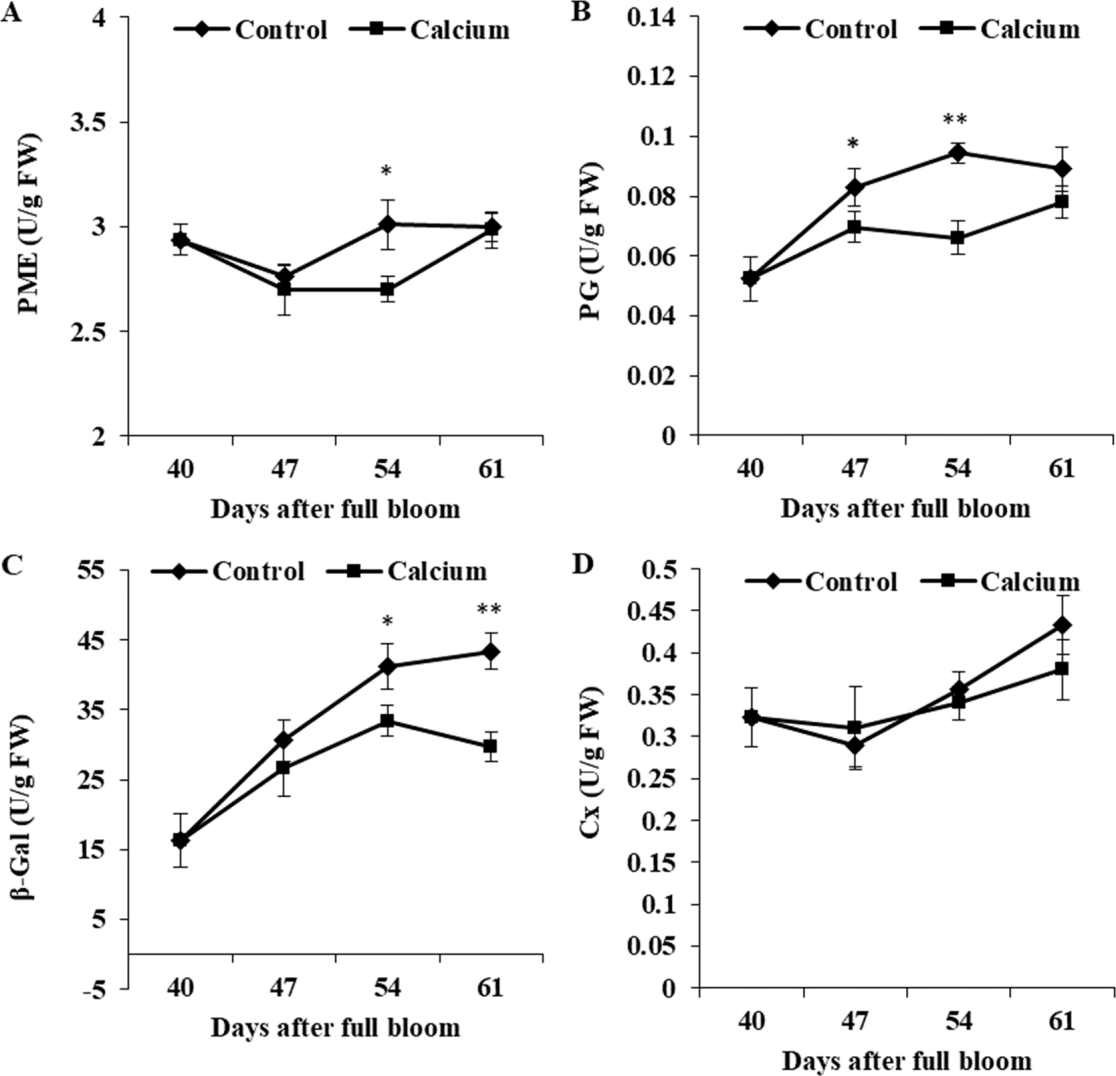
The activity of enzymes related to cell wall metabolism of calcium-treated and untreated grape skin. (A) Pectinmethylesterase. (B) Polygalacturonase. (C) *β*-galactosidase. (D) Cellulase. Error bar stands for standard deviation (SD). Asterisks “*” and “**” indicate significant difference between calcium and control at *P* < 0.05 and *P* < 0.01 level, respectively.

Activity of PME in the control initially decreased from 40 to 47 DAFB and thereafter increased and peaked at 54 DAFB. Calcium treatment inhibited the increase in PME activity, which peaked at 61 DAFB. Activity of PG was maintained at a constant level from 47 to 61 DAFB, and activity in the calcium treatment was significantly lower than that in the control. The *β*-Gal activity in the control increased steadily from 40 to 61 DAFB, whereas activity in the calcium treatment peaked at 54 DAFB followed by a distinct decline. Activity of *β*-Gal was inhibited in the calcium treatment from 47 to 61 DAFB. Activity of Cx changed little during ripening, and no significant difference between the calcium treatment and control was observed at all three time points (Fig. 13).

### Transcriptional analysis of genes involved in cell wall modification in the berry skin under calcium treatment

**Figure 14:**
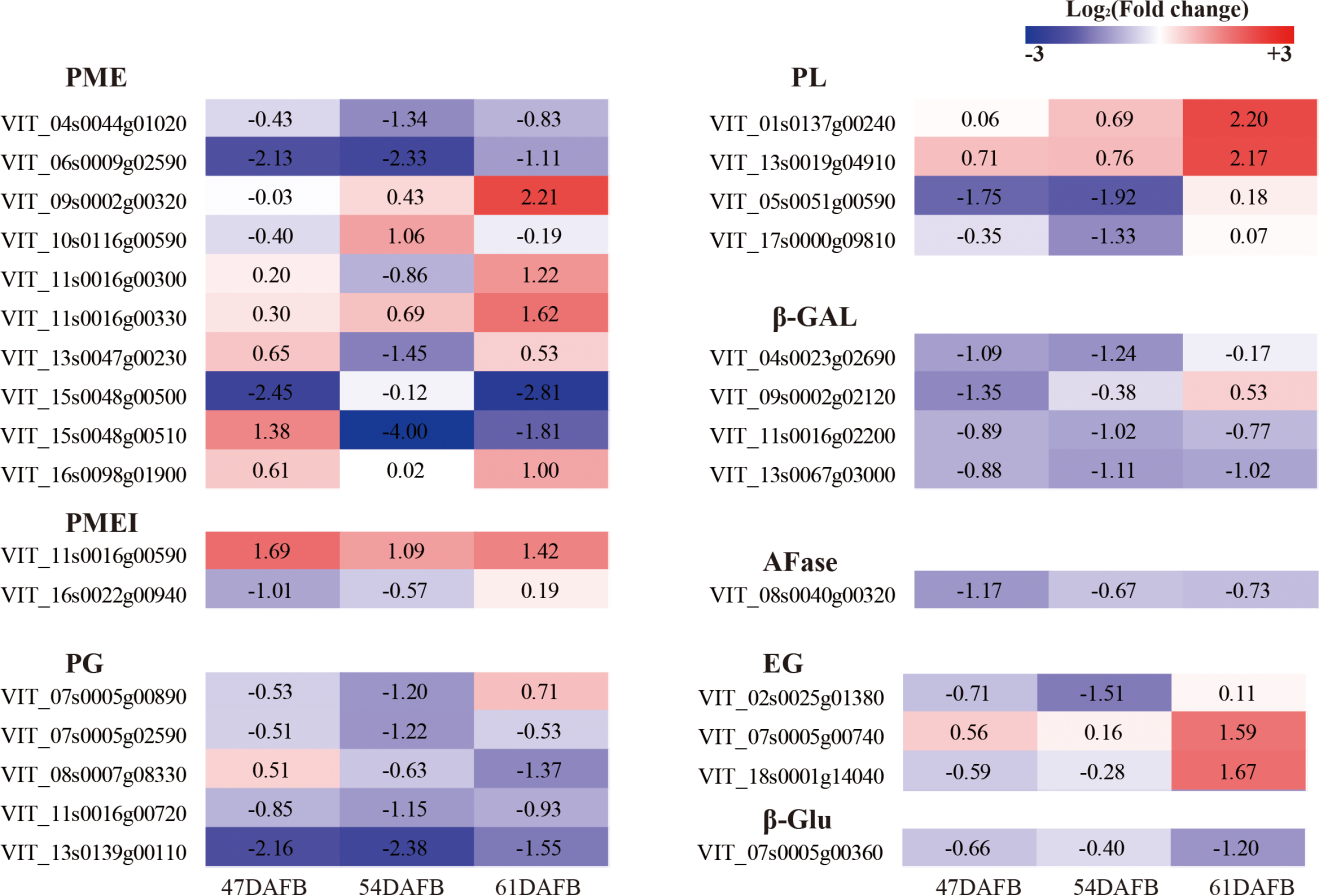
Heat map of log_2_ (Fold change) of the DEGs involved in cell wall metabolism.

To determine the genes involved in cell wall modification and regulated by calcium, the genes with FPKM <5 at all three time points were discarded and the genes defined as significant DEGs for at least one time point were selected for further analysis. We focused on genes associated with cell wall modification and identified ten *PME*, two *PMEI* (*PME inhibitor*), five *PG*, four *PL* (*pectate lyase*), four *β*-Gal, one *AFase* (*alpha-L-arabinofuranosidase*), three *EG* (*endoglucanase*), and one *β*-Glu (*Beta-glucosidase*) (Fig. 14 and Table S1). One *AFase* and almost all *β-Gal* genes were downregulated by calcium at all three time points except VIT_09s0002g02120 at 61 DAFB. Almost all *PG* genes were downregulated by calcium except VIT_07s0005g00890 at 61 DAFB and VIT_08s0007g08330 at 47 DAFB. Among the four *PL* genes, two were upregulated successively, whereas the other two genes were downregulated at 47 and 54 DAFB and upregulated at 61 DAFB. In particular, VIT_05s0051g00590, which showed the highest expression level and fold-changes, was significantly downregulated by calcium at 47 and 54 DAFB. Of the ten *PME* genes, two were significantly downregulated and one upregulated at 47 DAFB, four were significantly downregulated and one upregulated at 54 DAFB, and three were significantly downregulated and four upregulated at 61 DAFB. Only VIT_06s0009g02590 was continuously and significantly downregulated by calcium. With regard to *PMEI* genes, VIT_11s0016g00590 was continuously and significantly upregulated by calcium, whereas VIT_16s0022g00940 was significantly downregulated only at 47 DAFB. Four genes involved in cellulose degradation, consisting of three *EG* genes and one *β*-Glu, were identified. Three of these four genes were downregulated from 47 to 54 DAFB; only VIT_02s0025g01380 was significantly regulated by calcium. At 61 DAFB, two significantly upregulated *EG* and one significantly downregulated *β-Glu* gene were detected.

## Discussion

The goal of our study was to evaluate how calcium treatment affects fruit cracking and to elucidate the underlying physiological and molecular mechanism. We studied the effect of exogenous calcium on grape berry cracking, calcium absorbance and distribution, and cell wall metabolism in the cracking-susceptible cultivar ‘Xiangfei’. We investigated these indicators from 40 to 61 DAFB because (1) during this sampling period, the increasing difference in fruit-cracking incidence between the control and treatment (i.e., changed from 0.16% to 54.15%) best represents their dynamic gene expression profiles and most newly formed cracks may not attract that many microorganisms; and (2) at 68 and 75 DAFB, the differences in fruit-cracking incidence between the control and treatment remained constant (Fig. S3). During these later observation periods, previously formed cracks may attract microorganisms that could influence cell wall metabolism (Chardonnet, L’Hyvernay & Doneche, 1997; Kubicek, Starr & Glass, 2014). Therefore, as the most representative sampling period and to decrease the confounding influence of microorganisms, the subcellular, physiological, biochemical, and transcriptional analyses were only conducted from 40 to 61 DAFB. The results suggested that dipping fruit with 5 g L^−1^ CaCl_2_ effectively enhanced calcium pools in the vacuole and cell wall, inhibited production of WSP, and delayed degradation of protopectin (CSP and SSP) by regulating genes and enzymes associated with cell wall disassembly, enhanced the skin break force and, ultimately, significantly reduced the frequency and degree of fruit cracking.

### Dipping fruit with 5 g L^−1^ CaCl_2_ is effective to reduce fruit cracking

It is widely accepted that foliar sprays of calcium solutions have a positive effect in preventing fruit cracking in sweet cherry (Erogul, 2014; Kafle et al., 2016; Michailidis et al., 2017), pomegranate (Bakeer, 2016; Sheikh & Manjula, 2012) and navel orange (Chen et al., 2014; Wen & Shi, 2012). The present study showed that dipping grape berries in calcium solution also significantly reduced fruit cracking and the effect was superior to that of foliar sprays (Table S1). By determining the cracking severity index and observing micrographs of the berry surface, we established that calcium reduced the crack length, width, and depth (Figs. 4 and 5).

In sweet cherry, incubation of detached fruit with CaCl_2_ or FeCl_3_ reduces fruit cracking, and cracks in the FeCl_3_ treatment were consistently shallower than those in water-treated fruit (Weichert et al., 2004). In the present study, the reduced depth and extent of cracks in the calcium treatment (Fig. 5) may be explained by increased cross linking of homogalacturonans by di- and trivalent ions (Schumann et al., 2019; Weichert et al., 2004).

### Dipping fruit with calcium solution may stimulate Ca accumulation at stem-end region

In grape berries, the calcium content does not normally increase after veraison owing to the nonfunctional xylem, low mobility of calcium in the phloem, and low transpiration rate of the fruit (Knipfer et al., 2015; Rogiers et al., 2006; Rogiers et al., 2000). Therefore, to prevent localized calcium deficiency and promote fruit quality, it is necessary to supply calcium during the ripening stage. In the present study, atomic absorption spectrometry (Fig. 7), X-ray microanalysis (Fig. 8), and potassium-pyroantimonate precipitation (Figs. 9 and 10) revealed that calcium absorption was significantly higher in the calcium treatment compared with the control. In apple, the absorption efficiency of calcium is about 30% after brushing ^44^CaCl_2_ directly onto fruit (Kalcsits et al., 2017). In citrus, the absorption rate of ^45^Ca is 77% at 128 h after calcium treatment (Xiao et al., 1996). Thus, applying calcium directly to fruit is an effective method to supplement calcium nutrition.

In the present study, the absorbed calcium may be mainly absorbed by berry cells at the stem-end region. Becker & Knoche (2011) reported that water uptake through the stem surface and stem/fruit juncture accounted for about 80% of the total water absorbed by an immersed grape berry (Becker & Knoche, 2011). Although these authors studied water uptake, the data are also applicable to calcium penetration. After immersion of grape berry in fluorescent dye solution, the dye was mainly distributed at the stem-end of the berry and in vascular bundles of the brush region (Becker, Grimm & Knoche, 2012), which suggests that in the current study the absorbed calcium may have been largely transported to the stem-end region. As is shown in Fig. 1, ‘Xiangfei’ berries were characterized by concentric cracking at the stem-end region. Thus, we inferred that a high calcium content in stem-end region may play an important role in preventing the occurrence of concentric cracking.

### Calcium may reduce fruit cracking by enhancing Ca^2+^ pools

Calcium and boron are important elements required for cell wall strengthening, thereby influencing the mechanical properties of plant tissues (Aghdam et al., 2012; Dodd, 2010; Shi et al., 2017; White & Broadley, 2003). Calcium and boron deficiencies may increase fruit susceptibility to cracking (Huang et al., 1999; Khadivi-Khub, 2014). Calcium is the richest mineral element in plant cell wall, accounting for 60%–75% of the total tissue calcium content (Aghdam et al., 2012). Calcium crosslinks the carboxyl groups of the homogalacturonan domain and forms an “egg-box” structure, resulting in a stronger cell wall (Shi et al., 2017; Yang et al., 2016). Boron bridges two apiosyl residues of rhamnogalacturonan II side chains and increases the stability of calcium bridges (Shi et al., 2017). Foliar application of calcium and boron separately or in combination can lead to a significant decrease in fruit cracking in pomegranate and litchi (Ghanbarpour, Rezaei & Lawson, 2019; Ihsanul & Rab, 2012). In litchi the pericarp of cracked fruit contains lower boron and calcium contents than that of noncracked fruit, and fruit with a higher calcium content are more resistant to cracking (Huang et al., 2001; Li & Huang, 1996). The contents of calcium and boron are significantly higher, whereas the potassium and magnesium contents are markedly lower, in the pericarp of cracking-resistant tomato cultivars compare with those of susceptible cultivars (Yang et al., 2016). Magnesium and potassium can compete with calcium for binding sites and inhibit calcium absorbance, therefore the nutrient concentration ratios are generally used as indicators to predict the ability of a fruit to utilize calcium (Falchi et al., 2017). In tomato, the correlation between cracking and Ca/(Ca+Mg+K) is significantly higher than that between single or two ions and cracking (Yang et al., 2016). In the present study, compared with the control, calcium treatment resulted in higher calcium and boron contents (Fig. 7), and a higher Ca/(Ca+Mg+K) ratio (Fig. S4), which suggested that calcium may reduce fruit cracking by increasing the calcium and boron contents in the fruit pericarp and strengthening the cell wall structure. At the subcellular level, dipping fruit in calcium solution enhanced localization of calcium grains in the vacuole and cell wall (Fig. 11), which are the two predominant calcium pools and maintain calcium homeostasis at a calcium-deficient stage (Zhi et al., 2017).

### Calcium may reduce fruit cracking by inhibiting cell wall disassembly

The plant cell wall is a complex network composed of diverse polysaccharides (pectins, cellulose, and hemicellulose), proteins, and phenolic compounds (Brummell, 2006; Yakushiji, Sakurai & Morinaga, 2001). Especially in fruit, the cell wall is highly enriched in pectins, which constitutes 50% of the cell wall mass (Brummell, 2006). In tomato, protopectins (SSP and CSP) and hemicellulose were more abundant in fruit of cracking-resistant ‘LA1698’ than in cracking-susceptible ‘LA2683’; however, the cellulose content in both genotypes showed no significant difference (Yang et al., 2016). In jujube fruit, decrease in protopectin and cellulose and increase in WSP may contribute to the elevated cracking frequency during maturation (Wang et al., 2013). In the present study, calcium treatment significantly delayed the increase in WSP and the decrease in CSP and SSP contents (Fig. 12), which might contribute to stronger skin break force (Fig. 6) and higher resistance to fruit cracking (Fig. 4).

The changes in pectic cell wall components induced by calcium do not appear to be associated with delayed ripening, as no visible difference in ripening was observed between calcium-treated and control grape berries (Fig. 3). Many studies have shown that preharvest and postharvest calcium treatment can effectively delay post-harvest ripening and softening of fruit and extend the shelf life (Chéour et al., 1990; Ferguson, 1984; Gao et al., 2020; Hussain et al., 2012). However, how calcium affect ripening process of fruit on the tree is largely unexplored. Ortiz et al. reported that calcium applied as a foliar spray increases apple fruit firmness and prevents cell wall disassembly without influencing ethylene production and delaying fruit ripening (Ortiz, Graell & Lara, 2011). Another study also reported that elevated calcium content in apple fruit reduces the respiration rate without influencing the timing of the climacteric rise, leading to delayed senescence but not delayed maturation (Bramlage, Drake & Baker, 1974; Ferguson, 1984).

At the transcript level, *EG* and *β*-Glu transcription was not significantly regulated by calcium from 47 to 54 DAFB (Fig. 14), which coincided with the changes in cellulose and hemicellulose contents. Pectins are degraded by PME, PL, and PG (Wang et al., 2018). PME catalyzes the dimethyl-esterification of pectins and provides the substrate for PG and PL activities (Brummell, 2006; Deng, Wu & Li, 2005). Higher PG and PME activities and expression levels lead to fruit cracking (Brummell & Harpster, 2001; Li, Huang & Huang, 2003; Li et al., 2014; Lu & Lin, 2011). Silencing *SlPG* decreases the frequency of fruit cracking in transgenic tomato (Schuch et al., 1991). In the present study, the activities of PME and PG were strongly inhibited by calcium (Fig. 13). Transcript analysis revealed that one *PMEI* gene (VIT_11s0016g00590, PME inhibitor) was significantly up-regulated at all three time points by calcium (Fig. 14), which may inhibit *PME* expression and activity of PME; almost all *PG* genes were downregulated continuously by calcium and one *PL* gene (VIT_05s0051g00590) that showed a high expression level was significantly downregulated at 47 and 54 DAFB. Pectic and hemicellulosic polysaccharides are highly branched with galactosyl and arabinosyl-rich side chains, so suppression of the activities of *β*-Gal and AFase can control cell wall porosity and restrict access of pectolytic enzymes to their substrates (Belge et al., 2017; Brummell & Harpster, 2001). In tomato, *β*-Gal activity was higher in cracking-susceptible ‘LA2683’ (Yang et al., 2016). In the current study, *β*-Gal activity was inhibited by calcium treatment (Fig. 13), and transcription of one *AFase* and almost all *β*-Gal genes was downregulated by calcium at all three time points (Fig. 14). Collectively, these results suggest that calcium might relieve fruit cracking by inhibiting cell wall disassembly.

### Calcium may reduce fruit cracking by inhibiting cell wall swelling

Recent studies have shown that macrocracks, microcracks, cell death, cell wall swelling, and mechanical properties of the fruit skin are closely related (Brüggenwirth & Knoche, 2017; Schumann et al., 2019). Cell death or bursting is accompanied by loss of cell turgor and release of organic acids (Simon, 1977). The released acid in the apoplast can lead to solubilization of pectin (Dumville & Fry, 2003) and loss of calcium in the cell wall complex (Winkler, Ossenbrink & Knoche, 2015), resulting in swelling of the cell wall (Brüggenwirth & Knoche, 2017; Grimm & Knoche, 2015). Cell wall swelling is a critical factor in decreasing the fracture force and elasticity of the fruit skin, and aggravating rain-induced fruit cracking (Brüggenwirth & Knoche, 2017). More importantly, the process of cell wall swelling can be inhibited by calcium, leading to an increase in skin fracture force (Brüggenwirth & Knoche, 2017). Thus, we speculate that calcium may inhibit cell wall swelling by enhancing the calcium pools and inhibiting cell wall disassembly, thereby enhancing the skin mechanical properties and reducing fruit cracking. However, further studies are needed to determine whether cell wall swelling is inhibited by calcium in grape.

## Conclusion

The data presented herein indicate that dipping fruit in calcium solution can decrease the frequency of grape berry cracking without affecting fruit quality. The decrease in cracking incidence and severity may reflect that exogenous calcium can stimulate calcium uptake, inhibit WSP production, delay degradation of protopectin, stabilize the cell wall structure, enhance the mechanical properties of the skin, and thus confer the fruit with greater resistance to cracking.

##  Supplemental Information

10.7717/peerj.9896/supp-1 Supplemental Information 1 Gene annotation, FPKM, and fold changes (Log _2_ ratio) of DEGs involved in cell wall metabolism

10.7717/peerj.9896/supp-2 Supplemental Information 2 Effect of different calcium concentration and application methods on fruit cracking incidence Different letters indicate significant difference at *P* < 0.05.

10.7717/peerj.9896/supp-3 Supplemental Information 3 Environmental variables during the experimental period The numbers on the *x*-axis represent 1 June to 30 June. Blue arrows indicate the sampling dates.

10.7717/peerj.9896/supp-4 Supplemental Information 4 Fruit-cracking incidence of grape berry from 40 to 75 DAFB in calcium treatment and control Error bar stands for Standard deviation (SD).

10.7717/peerj.9896/supp-5 Supplemental Information 5 The ratio of Ca/(Ca+K+Mg) of calcium-treated and untreated grape skin Error bar stands for Standard deviation (SD). Asterisks “*” and “**” indicate significant difference between calcium and control at* P* < 0.05 and *P* < 0.01 level, respectively.

10.7717/peerj.9896/supp-6 Supplemental Information 6 Raw data of the figures and tables in our manuscript

10.7717/peerj.9896/supp-7 Supplemental Information 7 FPKM and annotations of all detected genes

## Additional Information and Declarations

### DNA Deposition

The following information was supplied regarding the deposition of DNA sequences: All RNA-Seq raw data files are available at NCBI SRA: SRR11531447, SRR11531448, SRR11531449, SRR11531450, SRR11531451, SRR11531452.

### Data Availability

The following information was supplied regarding data availability: The raw data are available in the Supplementary Files.
