# Supplementary figures and images for: Effect of calcium on relieving berry cracking in grape (Vitis vinifera L.) ‘Xiangfei’
Source: PeerJ. 2020 Sep 15;8:e9896. doi: 10.7717/peerj.9896 (PMC7500324; doi:10.7717/peerj.9896)

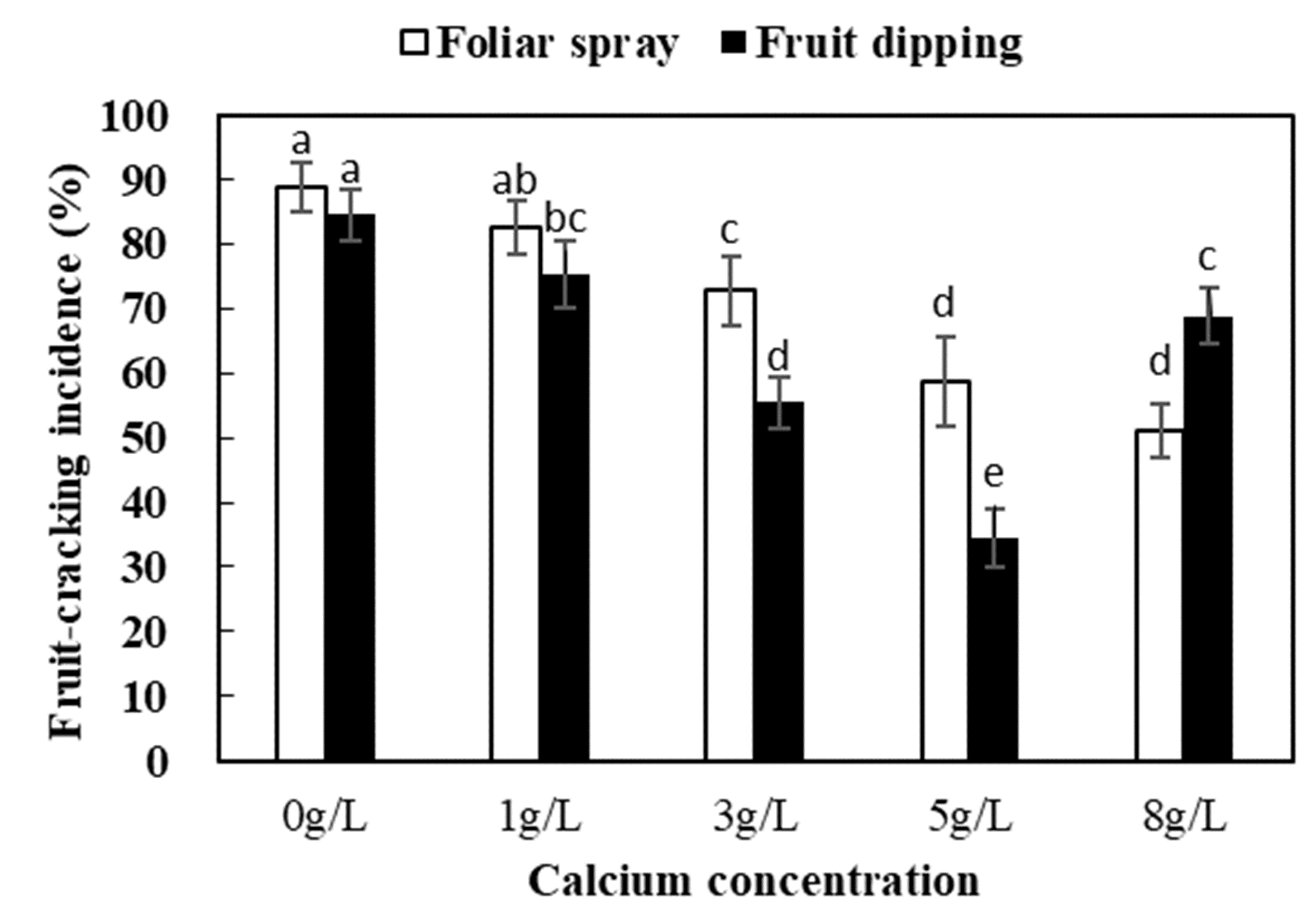

Supplement: Supplemental Information 2 — Different letters indicate significant difference at P < 0.05. [file peerj-08-9896-s002.png]

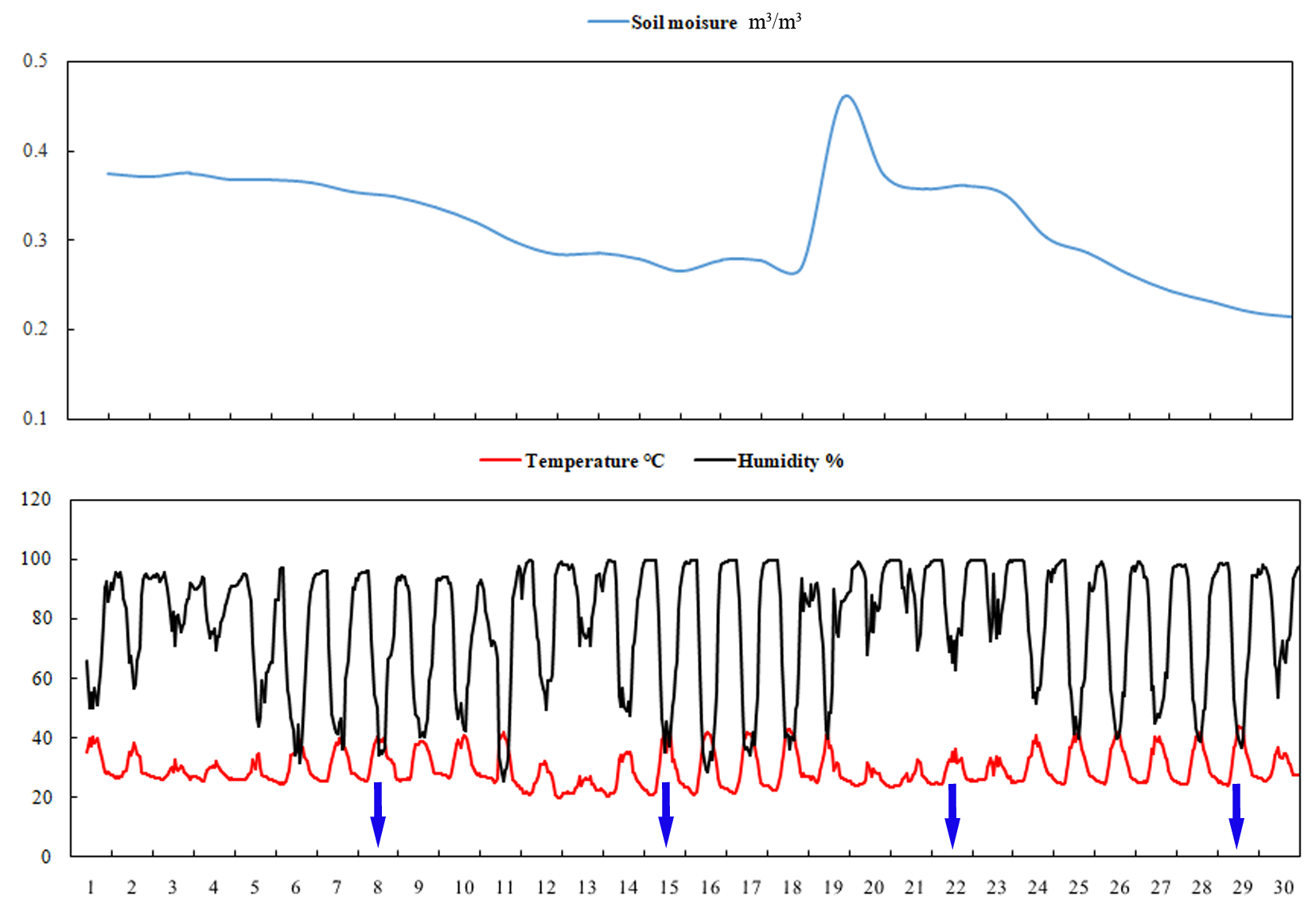

Supplement: Supplemental Information 3 — The numbers on the x-axis represent 1 June to 30 June. Blue arrows indicate the sampling dates. [file peerj-08-9896-s003.png]

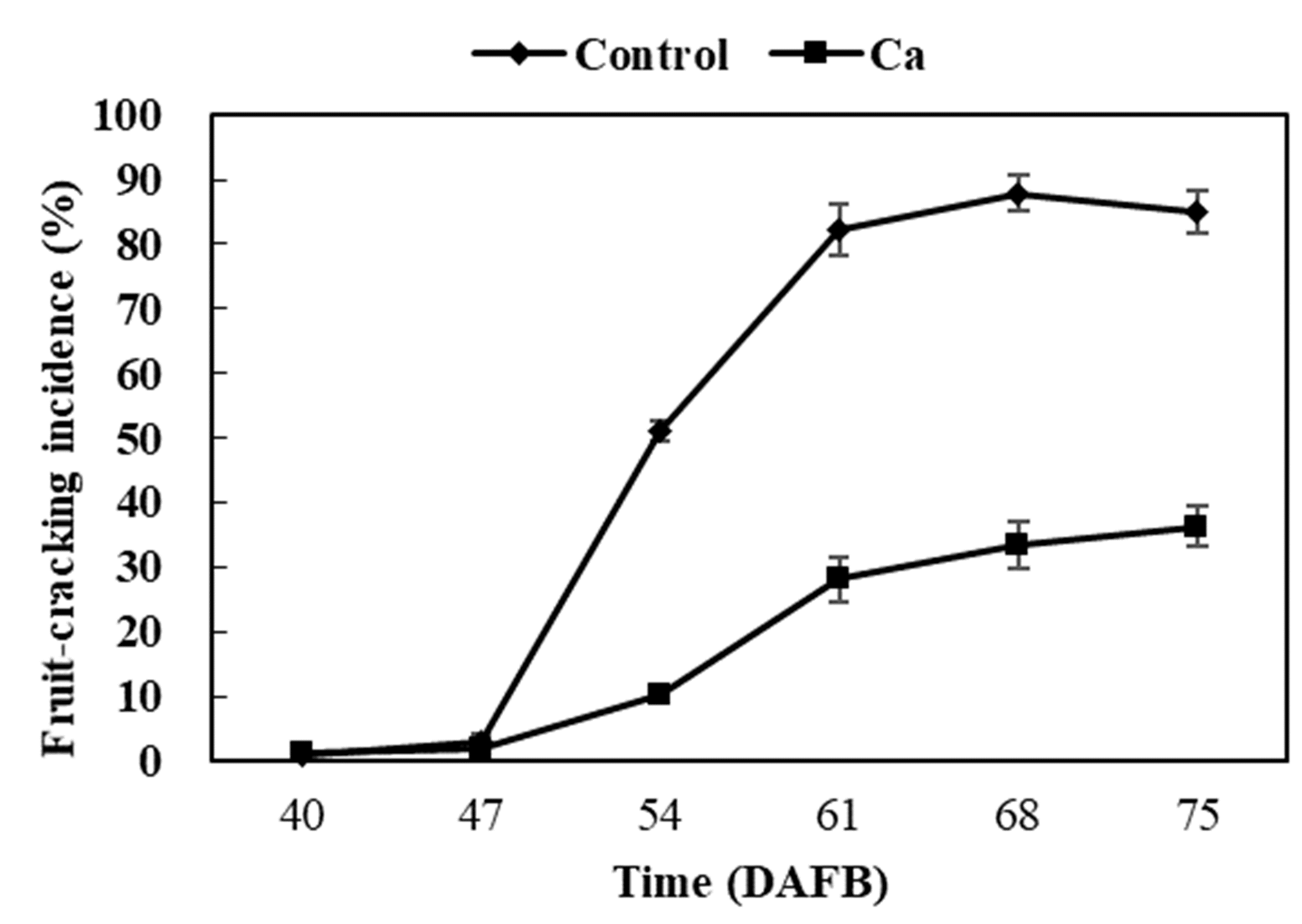

Supplement: Supplemental Information 4 — Error bar stands for Standard deviation (SD). [file peerj-08-9896-s004.png]

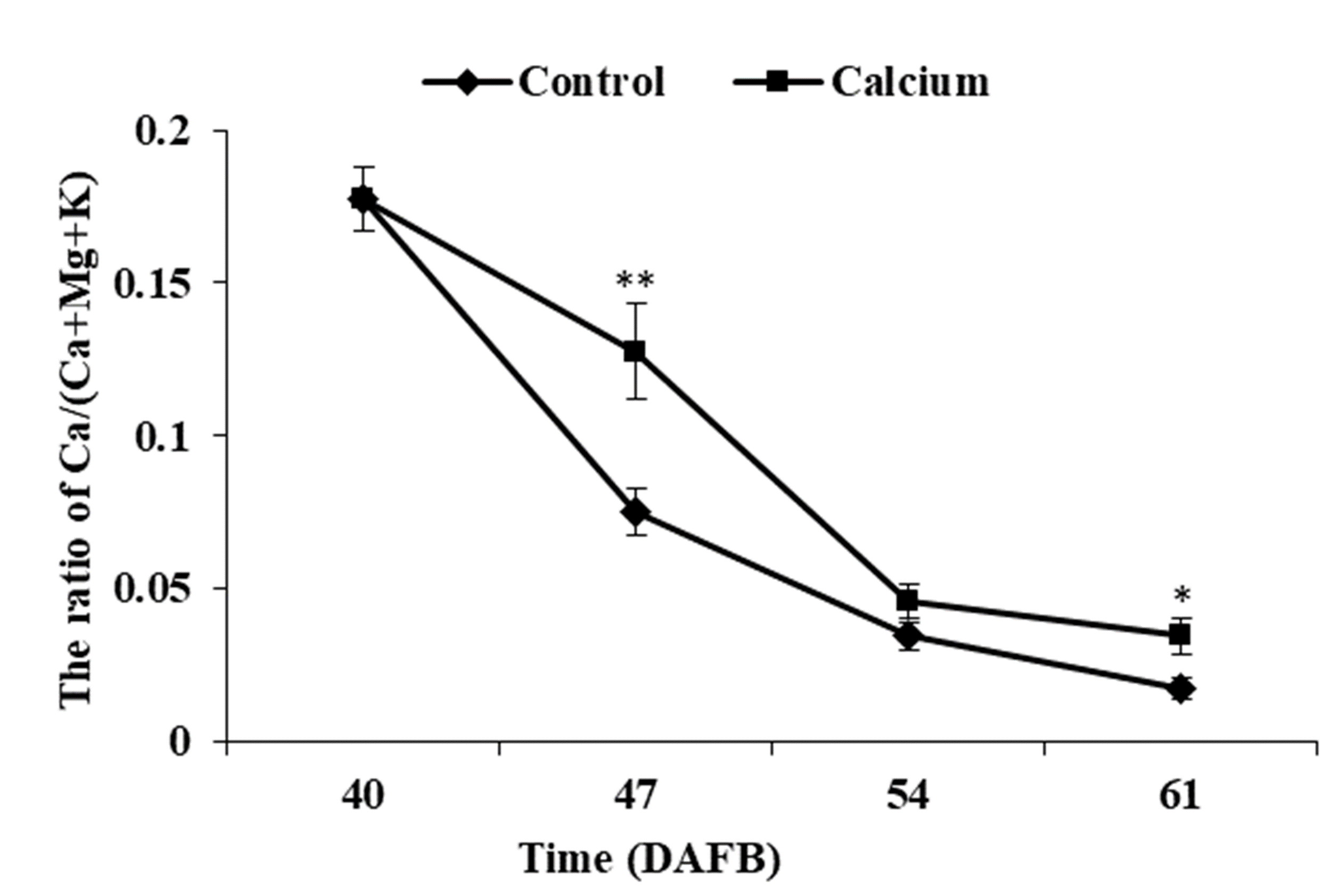

Supplement: Supplemental Information 5 — Error bar stands for Standard deviation (SD). Asterisks “*” and “**” indicate significant difference between calcium and control at P < 0.05 and P < 0.01 level, respectively. [file peerj-08-9896-s005.png]
